# cDNA-AFLP analysis reveals the adaptive responses of citrus to long-term boron-toxicity

**DOI:** 10.1186/s12870-014-0284-5

**Published:** 2014-10-28

**Authors:** Peng Guo, Yi-Ping Qi, Lin-Tong Yang, Xin Ye, Huan-Xin Jiang, Jing-Hao Huang, Li-Song Chen

**Affiliations:** College of Resource and Environmental Science, Fujian Agriculture and Forestry University, Fuzhou, 350002 China; Institute of Horticultural Plant Physiology, Biochemistry and Molecular Biology, Fujian Agriculture and Forestry University, Fuzhou, 350002 China; Institute of Materia Medica, Fujian Academy of Medical Sciences, Fuzhou, 350001 China; College of Life Science, Fujian Agriculture and Forestry University, Fuzhou, 350002 China; Institute of Fruit Tree Science, Fujian Academy of Agricultural Sciences, Fuzhou, 350013 China; Fujian Key Laboratory for Plant Molecular and Cell Biology, Fujian Agriculture and Forestry University, Fuzhou, 350002 China; The Higher Educational Key Laboratory of Fujian Province for Soil Ecosystem Health and Regulation, Fujian Agriculture and Forestry University, Fuzhou, 350002 China

**Keywords:** Boron-tolerance, Boron-toxicity, cDNA-AFLP, *Citrus grandis*, *Citrus sinensis*, Photosynthesis

## Abstract

**Background:**

Boron (B)-toxicity is an important disorder in agricultural regions across the world. Seedlings of ‘Sour pummelo’ (*Citrus grandis*) and ‘Xuegan’ (*Citrus sinensis*) were fertigated every other day until drip with 10 μM (control) or 400 μM (B-toxic) H_3_BO_3_ in a complete nutrient solution for 15 weeks. The aims of this study were to elucidate the adaptive mechanisms of citrus plants to B-toxicity and to identify B-tolerant genes.

**Results:**

B-toxicity-induced changes in seedlings growth, leaf CO_2_ assimilation, pigments, total soluble protein, malondialdehyde (MDA) and phosphorus were less pronounced in *C. sinensis* than in *C. grandis*. B concentration was higher in B-toxic *C. sinensis* leaves than in B-toxic *C. grandis* ones. Here we successfully used cDNA-AFLP to isolate 67 up-regulated and 65 down-regulated transcript-derived fragments (TDFs) from B-toxic *C. grandis* leaves, whilst only 31 up-regulated and 37 down-regulated TDFs from B-toxic *C. sinensis* ones, demonstrating that gene expression is less affected in B-toxic *C. sinensis* leaves than in B-toxic *C. grandis* ones. These differentially expressed TDFs were related to signal transduction, carbohydrate and energy metabolism, nucleic acid metabolism, protein and amino acid metabolism, lipid metabolism, cell wall and cytoskeleton modification, stress responses and cell transport. The higher B-tolerance of *C. sinensis* might be related to the findings that B-toxic *C. sinensis* leaves had higher expression levels of genes involved in photosynthesis, which might contribute to the higher photosyntheis and light utilization and less excess light energy, and in reactive oxygen species (ROS) scavenging compared to B-toxic *C. grandis* leaves, thus preventing them from photo-oxidative damage. In addition, B-toxicity-induced alteration in the expression levels of genes encoding inorganic pyrophosphatase 1, AT4G01850 and methionine synthase differed between the two species, which might play a role in the B-tolerance of *C. sinensis*.

**Conclusions:**

*C. sinensis* leaves could tolerate higher level of B than *C. grandis* ones, thus improving the B-tolerance of *C. sinensis* plants. Our findings reveal some novel mechanisms on the tolerance of plants to B-toxicity at the gene expression level.

**Electronic supplementary material:**

The online version of this article (doi:10.1186/s12870-014-0284-5) contains supplementary material, which is available to authorized users.

## Background

Althought boron (B) is a micronutrient element required for normal growth and development of higher plants, it is harmful to plants when present in excess. Whilst of lesser importance than B-deficiency (a widespread problem in many agricultural crops), B-toxicity is also an important problem in agricultural regions across the world, which citrus trees are cultivated [1-3]. Despite the importance of B-toxicity for crop productivity, the mechanisms by which plants respond to B-toxicity are poorly understood yet. Recently, increasing attention has been paid to plant B-toxicity as a result of the increased demand for desalinated water, in which the B level may be too high for healthy irrigation of crops [4].

Alteration of gene expression levels is an inevitable process of plants responding to environmental stresses. Kasajima and Fujiwara first investigated high B-induced changes in gene expression in *Arabidopsis thaliana* roots and rosette leaves using microarray, and identified a number of high B-induced genes, including a heat shock protein and a number of the multi-drug and toxic compound extrusion (MATE) family transporters [5]. Hassan et al. preformed suppression subtractive hybridization on root cDNA from bulked B-tolerant and -intolerant doubled haploid barley lines grown under moderate B-stress and identified 111 upregulated clones in the tolerant bulk under B-stress, nine of which were genetically mapped to B-tolerant quantitative trait loci. An antioxidative response mechanism was suggested to provide an advantage in tolerating high level of soil B [6]. Recently, Aquea et al. found that B-toxicity upregulated the expression of genes related to ABA signaling, ABA response and cell wall modification, and downregulated the expression of genes involved in water transporters in *Arabidopsis* roots, concluding that root growth inhibition was caused by B-toxicity-induced water-stress [7]. Most research, however, has focused on roots and herbaceous plants (i.e., barley, *A. thaliana*), very little is known about the differential expression of genes in response to B-toxicity in leaves and woody plants.

Citrus belongs to evergreen subtropical fruit trees. In China, B-toxicity often occurs in citrus orchards from high level of B in soils and/or irrigation water and from inappropriate application of B fertilizer especially under low-rainfall conditions [8,9]. During 1998–1999, Huang et al. investigated the nutrient status of soils and leaves from 200 ‘Guanximiyou’ pummelo (*Citrus grandis*) orchards located in Pinghe, Zhangzhou, China. Up to 61.5% and 17.0% of orchards were excess in leaf B and soil water-soluble B, respectively [10]. Previous studies showed that B-toxicity disturbed citrus plant growth and metabolism in multiple way, including interference of nutrient uptake [2], ultrastructural damage of roots and leaves [11-13], inhibition of CO_2_ assimilation, photosynthetic enzymes and photosynthetic electron transport, decrease of chlorophyll (Chl), carotenoid (Car) and total soluble protein levels, affecting leaf carbohydrate metabolism and antioxidant system [9,14]. However, our understanding of the molecular mechanisms underlying these processes in citrus is very limited. To our best knowledge, no high B-toxicity-induced changes in gene expression profiles have been reported in citrus plants to date. Here we investigated the effects of B-toxicity on growth, leaf CO_2_ assimilation, leaf concentrations of malondialdehyde (MDA), pigments and total soluble protein, root and leaf concentration of B, leaf concentration of phosphorus (P), and leaf gene expression profiles using cDNA-amplified fragment length polymorphism (cDNA-AFLP) in *Citrus grandis* and *Citrus sinensis* seedlings differing in B-tolerance [13]. The aims of this study were to elucidate the adaptive mechanisms of citrus plants to B-toxicity and to identify B-tolerant genes.

## Results

### Effects of B-toxicity on seedlings growth, B concentration in roots and leaves, and P concentration in leaves

Because B is phloem immobile in citrus plants, B-toxic symptoms first developed in old leaves. The typical visible symptom produced in B-toxic leaves was leaf burn (chlorotic and/or necrotic), which only occurred in *C. grandis* plants. In the later stages, B-toxic leaves shed premature. By contrast, almost no visible symptoms occurred in *C. sinensis* plants except for very few plants (Additional file 1).

**Figure 1:**
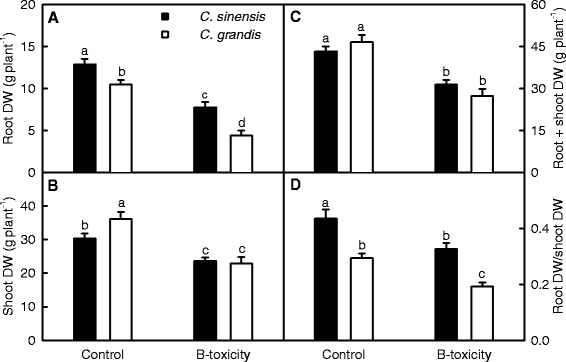
**Effects of B**-**toxicity on growth of** ***Citrus sinensis*** **and** ***C. grandis*** **seedlings.** Bars represent means ± SE (*n* =10). **(A** **-C)** Root, shoot and root + shoot DWs. **(D)** Ratio of root DW to shoot DW. Bars represent means ± SE (*n* =10). Different letters above the bars indicate a significant difference at *P* <0.05.

B-toxicity-induced decreases in root, shoot and whole plant dry weights (DWs) were more pronounced in *C. grandis* than in *C. sinensis* seedlings (Figure 1A-C). Root DW decreased to a larger extent than shoot DW in response to B-toxicity, and resulted in a decrease in root DW/shoot DW ratio of both *C. grandis* and *C. sinensis* seedlings (Figure 1A-B and D).

**Figure 2:**
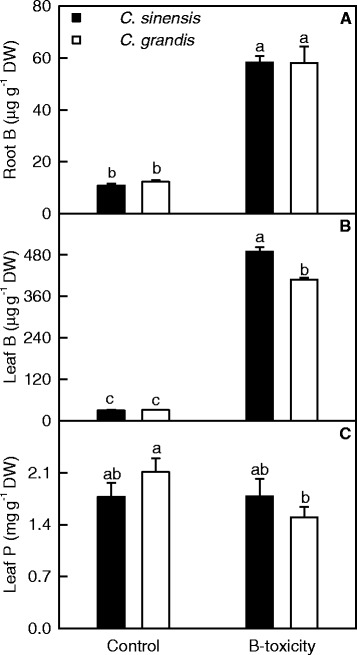
**Effects of B**-**toxicity on root and leaf B and leaf P.** **(A-** **B)** Root and leaf B concentration. **(C)** Leaf P concentration. Bars represent means ± SE (*n* =4 or 5). Different letters above the bars indicate a significant difference at *P* <0.05.

B-toxicity increased B concentration in roots and leaves, especially in leaves and decreased P concentration in *C. grandis* leaves. No significant differences were found in root and leaf B concentration and leaf P concentration between the two species at each given B treatment except that B concentration was higher in B-toxic *C. sinensis* leaves than in B-toxic C. *grandis* ones (Figure 2).

### Effects of B-toxicity on leaf gas exchange, pigments, total soluble protein and MDA

**Figure 3:**
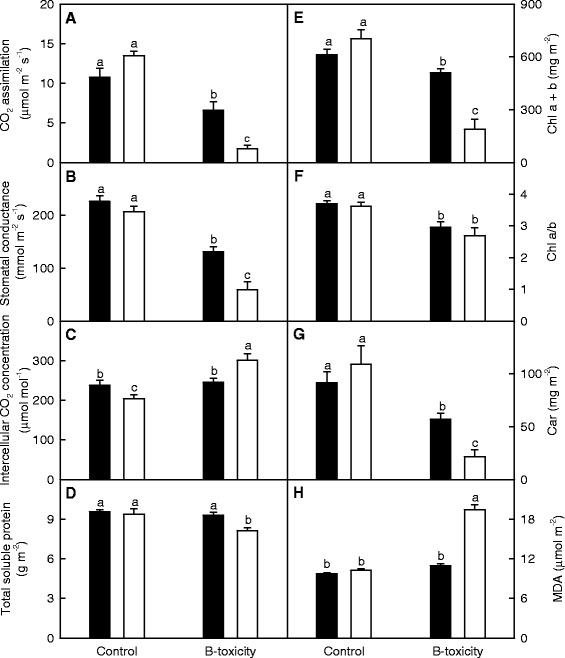
**Effects of B**-**toxicity on leaf gas exchange**, **total soluble protein**, **pigments and MDA.** **(A-** **C)** CO_2_ assimilation, stomatal conductance and intercellular CO_2_ concentration. **(D)** Total soluble protein concentration. **(E)** Chl a + b concentration. **(F)** Chl a/b ratio. **(G)** Car concentration. **(H)** MDA concentration. Bars represent means ± SE (*n* =4 or 5). Different letters above the bars indicate a significant difference at *P* <0.05.

B-toxicity-induced decreases in both CO_2_ assimilation and stomatal conductance were higher in *C. grandis* than in *C. sinensis* leaves. Intercellular CO_2_ concentration increased in *C. grandis* leaves, but did not significantly change in *C. sinensis* leaves in response to B-toxicity. CO_2_ assimilation and stomatal conductance in control leaves did not differ between the two species, but were higher in B-toxic *C. sinensis* leaves than in B-toxic *C. grandis* ones. Intercellular CO_2_ concentration in control leaves was higher in *C. sinensis* than in *C. grandis*, but the reverse was the case in B-toxic leaves (Figure 3A-C).

B-toxicity decreased concentrations of Chl a + b and Car and ratio of Chl a/b in *C. grandis* and *C. sinensis* leaves. In control leaves, all the three parameters did not differ between the two species, but Chl a + b and Car concentrations were higher in B-toxic *C. sinensis* leaves than in B-toxic *C. grandis* ones (Figure 3E-G).

Leaf concentrations of total soluble protein and MDA were decreased and increased by B-toxicity in *C. grandis* leaves, respectively, but were not significantly affected in *C. sinensis* ones (Figure 3D and H).

### B-toxicity-induced differentially expressed genes revealed by cDNA-AFLP

Here we used a total of 256 selective primer combinations to isolate the differentially expressed transcript-derived fragments (TDFs) from B-toxic leaves of two citrus species differing in B-tolerance. A representative picture of a silver-stained cDNA-AFLP gel showing B-toxicity-induced genes in *C. grandis* and *C. sinensis* leaves was presented in Additional file 2. As shown in Table 1, a total of 6050 clear and unambiguous TDFs were detected from the B-toxic leaves, with an average of 25.7 (15–40) TDFs for each primer combination. Among these TDFs, 932 TDFs only presented in *C. grandi*s, 631 TDFs only presented in *C. sinensis*, and 4587 TDFs presented in the two species.

**Table 1:** **Summary of transcript**-**derived fragments** (**TDFs**) **from control and boron** (**B**)-**toxic leaves of** ***Citrus grandis*** **and** ***Citrus sinensis***

	**Number of TDFs**			
	**Only present in** ***C. grandis***	**Only present in** ***C. sinensis***	**Present in both species**	**Total**
Total TDFs detected from gels	932	631	4487	6050
Total differentially expressed TDFs recovered from gels	164	50	54	268
TDFs produced useable sequence data	139	46	44	229
TDFs encoding known or putative proteins	97	40	23	160
TDFs encoding predicted, uncharacterized, hypothetical or unnamed proteins	9	2	3	14
TDFs without database matches	33	4	18	55

**Figure 4:**
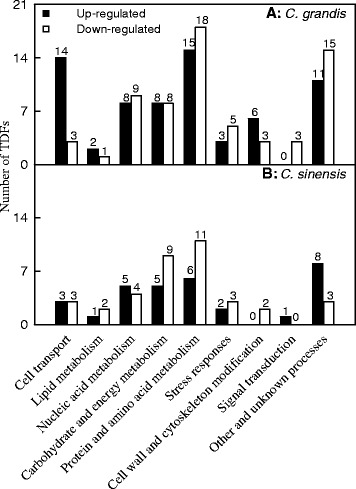
**Functional classification of differentially expressed TDFs under B**-**toxicity in** ***Citrus grandis*** **(A)** **and** ***Citrus sinensis*** **leaves** **(B).** Functional classification was performed based on the information reported for each sequence by The Gene Ontology (http://amigo1.geneontology.org/cgi-bin/amigo/blast.cgi) and Uniprot (http://www.uniprot.org/).

A total of 218 and 104 differentially expressed and reproducible TDFs were successfully obtained from B-toxic *C. grandis* and *C. sinensis* leaves, respectively. All these TDFs were re-amplified, cloned and sequenced. For *C. grandis*, 183 of fragments yielded usable sequence data. Aligment analysis showed 132 TDFs were homologous to genes encoding known, putative predicted, uncharacterized, hypothetical or unnamed proteins, and the remaining 51 TDFs showed no significant matches (Tables 1 and 2). Among these matched TDFs, 67 (50.8%) TDFs were up-regulated and 65 (49.2%) were down-regulated by B-toxicity. These TDFs were related to different biological processes such as cell transport (12.9%), lipid metabolism (2.3%), nucleic acid metabolism (12.9%), carbohydrate and energy metabolism (12.1%), protein and amino acid metabolism (25.0%), stress responses (6.1%), cell wall and cytoskeleton modification (6.8%), signal transduction (2.3%), other and unknown processes (19.7%) (Figure 4A). For *C. sinensis* leaves, 90 differentially expressed TDFs produced readable sequences (Tables 1 and 2), 68 of which displayed homology to genes encoding known, putative, hypothetical, uncharacterized or unnamed proteins. The remaining 22 TDFs had no database matches. Of these matched TDFs, 31 (45.6%) TDFs increased and 37 (54.4%) decreased in response to B-toxicity. These TDFs were involved in cell transport (8.8%), lipid metabolism (4.4%), nucleic acid metabolism (13.2%), carbohydrate and energy metabolism (20.6%), protein and amino acid metabolism (25.0%), stress responses (7.4%), cell wall and cytoskeleton modification (2.9%), signal transduction (1.5%), other and unknown processes (16.2%) (Figure 4B).

**Table 2:** **Homologies of differentially expressed cDNA**-**AFLP fragments with known gene sequences in database using BLASTN algorithm along their expression patterns in B**-**toxic leaves of** ***Citrus grandis*** **and** ***Citrus sinensis***

**TDF #**	**Size** **(bp)**	**Homology**	**Organism origin**	**E**-**value**	**Similarity (%)**	**Genebank ID**	**Ratio of BT/** **CK**	
							**CG**	**CS**
***Carbohydrate and energy metabolism***								
143_2	280	Ribulose-1,5-bisphosphate carboxylase/oxygenase small subunit precursor	*Citrus reticulata*	6.00E-49	93%	AAG49562.1	0	
251_1	329	Photosystem II 32 kDa protein (psbA)	*Dumortiera hirsuta*	1.00E-64	97%	AEI72217.1	0	
112_2	173	Chloroplast photosystem II oxygen-evolving complex 23 kDa polypeptide	*Cucumis sativus*	1.00E-18	75%	ABK55671.1	0	2.9
239_4	223	NifU-like protein	*Medicago truncatula*	3.00E-17	87%	XP_003594958.1	0	
23_2	253	Glyceraldehyde-3-phosphate dehydrogenase B	*Arabidopsis thaliana*	3.00E-06	84%	NP_174996.1		+
6_4	222	Rubisco activase	*A. thaliana*	1.00E-33	94%	BAF01986.1		0
249_3	313	Sedoheptulose-1 7-bisphosphatase	*M. truncatula*	2.00E-48	97%	XP_003600853.1	+	+
235_2	305	ADP-glucose pyrophosphorylase	*Pisum sativum*	5.00E-39	82%	CAA69978.1		0
42_1	193	Starch branching enzyme I	*Ipomoea batatas*	1.00E-27	90%	BAE96953.1	0	
59_2	287	Glucose-1-phosphate adenylyltransferase large subunit 1	*A. thaliana*	2.00E-32	77%	NP_197423.1	0	0
75_2	221	Citrate synthase	*Citrus maxima*	4.00E-34	97%	ADZ05826.1	2.8	
87_1	224	Pyruvate dehydrogenase E1 component subunit beta	*M. truncatula*	1.00E-26	83%	XP_003620963.1	+	
33_2	289	Aconitate hydratase 3	*Citrus clementina*	7.00E-50	94%	CBE71057.1	+	
161_3	257	2,3-bisphosphoglycerate- independent phosphoglycerate mutase	*Vitis amurensis*	1.00E-40	91%	ACI96093.1		+
35_1	160	Plastidial pyruvate kinase 3	*A. thaliana*	6.00E-21	96%	NP_564402.1		0
130_1	272	Aconitate hydratase 1	*Citrus clementina*	2.00E-31	98%	CBE71056.1		0
171_2	328	Protochlorophyllide oxidoreductase C (PORC, AT1G03630)	*A. thaliana*	1.00E-43	89%	BAH57125.1	0	0
5_1	192	Cytochrome P450	*Citrus sinensis*	2.00E-16	63%	AAL24049.1	+	
76_1	261	Cytochrome P450 like protein	*A. thaliana*	3.00E-29	68%	BAE99553.1	+	
237_2	258	1,3-beta-D-glucanase GH17_65	*Populus tremula* × *Populus tremuloides*	2.00E-31	78%	ADW08745.1		0
233_5	216	Alpha-glucan water dikinase 1	*A. thaliana*	4.00E-14	82%	NP_563877.1	+	0
57_3	176	UDP-D-glucuronate 4-epimerase 3	*A. thaliana*	1.00E-21	90%	NP_191922.1	0.3	+
117_2	242	Rubredoxin family protein	*A. thaliana*	8.00E-24	81%	NP_568342.1		0
121_1	179	Rieske iron-sulphur protein precursor	*Pinellia ternata*	6.00E-20	86%	CAM57108.1	+	
***Lipid metabolism***								
10_1	282	Fatty acid hydroperoxide lyase	*Citrus aurantium*	2.00E-41	100%	ABI64149.1		0
233_3	217	3-oxoacyl-reductase	*Zea mays*	2.00E-05	85%	NP_001167684.1		0
195_1	321	Sugar-dependent1	*Arabidopsis lyrata subsp. lyrata*	3.00E-28	86%	XP_002871068.1		+
8_1	232	Acyl carrier protein 1, chloroplastic-like	*Vitis vinifera*	6.4	42%	XP_003631979.1	0.4	
194_1	256	Alpha/beta-hydrolase domain-containing protein	*A. thaliana*	2.00E-34	72%	NP_181474.2	+	
186_4	276	Phospholipase-like protein (PEARLI 4) domain-containing protein	*A. thaliana*	7.00E-10	35%	NP_973499.1	+	
***Nucleic acid metabolism***								
52_1	248	Spliceosomal protein U1A	*A. thaliana*	1.00E-24	69%	NP_182280.1	+	
49_1	337	Heat stress transcription factor B-2b	*M. truncatula*	8.00E-32	78%	XP_003611134.1	+	
72_4	171	Global transcription factor gro + A2	*A. thaliana*	2.00E-04	69%	NP_192575.3	+	
120_1	257	IAA13	*Solanum lycopersicum*	3.00E-30	67%	AEX00356.1	3.3	
44_1	307	Elongator complex protein 3	*A. thaliana*	4.00E-53	89%	NP_568725.1	+	
159_2	366	Flowering time control protein FPA (AT2G43410)	*A. thaliana*	0.14	32%	BAH56948.1	+	
164_1	285	ABA responsive element-binding protein	*Solanum torvum*	3.00E-10	84%	AFA37978.1	+	0
73_2	255	Regulator of ribonuclease-like protein	*M. truncatula*	2.00E-08	83%	XP_003593378.1	+	+
250_3	305	RNA recognition motif-containing protein	*A. thaliana*	7.00E-31	70%	NP_563946.1	0.4	2.7
157_2	256	RNA recognition motif-containing protein	*A. thaliana*	3.00E-28	76%	NP_188119.1	0	+
11_1	353	Putative RNA helicase MTR4	*A. thaliana*	1.00E-44	82%	NP_176185.1	0	
71_3	209	RNA helicase SDE3	*A. thaliana*	7.00E-24	71%	AAK40099.1	0	0
186_1	395	Chromodomain-helicase-DNA-binding protein	*M. truncatula*	9.00E-56	73%	XP_003625728.1		0
108_1	317	Receptor for activated C kinase 1B	*A. thaliana*	3.00E-40	87%	NP_175296.1		0
67_4	195	Sequence-specific DNA binding transcription factor	*A. thaliana*	5.2	47%	NP_566386.1	0	
60_1	333	AT5g24120/MLE8_4	*A. thaliana*	9.00E-37	63%	AAK74018.1		+
10_4	114	GRAS family transcription factor	*Populus trichocarpa*	2.00E-04	78%	XP_002310226.1	0	
22_3	248	MAF1-like protein	*Citrus sinensis*	2.00E-24	96%	AEV43358.1	0	
131_1	270	RNA-binding (RRM/RBD/RNP motifs) family protein	*A. thaliana*	7.00E-23	62%	NP_171616.1		7.3
104_1	234	Zinc finger CCCH domain-containing protein	*M. truncatula*	1.00E-04	43%	XP_003605843.1	0	
68_2	217	F14N23.20	*A. thaliana*	3.00E-27	83%	AAD32882.1	0.3	
***Protein and amino acid metabolism***								
236_1	312	Translation initiation factor IF-2, chloroplastic (AT1G17220)	*A. thaliana*	4.00E-45	85%	BAH20402.1	0	
117_4	174	Eukaryotic release factor 1-3	*Brassica oleracea var.botrytis*	3.00E-22	94%	ACZ71035.1	0	
93_3	193	EMB1241	*A.s lyrata subsp. lyrata*	5.00E-09	69%	XP_002873846.1	0.4	
73_3	201	Ankyrin repeat domain-containing protein	*M. truncatula*	5.00E-19	66%	XP_003614004.1	0.2	
179_4	274	50S ribosomal protein L15	*A. thaliana*	1.00E-18	80%	NP_189221.1	0	
105_1	216	30S ribosomal protein S17	*M. truncatula*	0.005	89%	XP_003604547.1	0	0
99_6	165	60S ribosomal protein L6, putative	*A. thaliana*	2.00E-18	93%	AAM65875.1	0	
186_2	224	60S ribosomal protein L4-1	*A. thaliana*	3.00E-52	90%	NP_001030663.1	0	
129_2	253	60S ribosomal protein L23	*A. thaliana*	2.00E-74	97%	NP_001189805.1	+	
161_1	221	60S ribosomal protein L10B	*Hevea brasiliensis*	3.00E-27	83%	ADR71273.1	+	
93_2	210	SHEPHERD	*A. thaliana*	2.00E-26	86%	BAB86368.1	+	
98_1	272	Chaperonin 20	*A. thaliana*	2.00E-37	81%	NP_197572.1		0
69_3	174	AT5G47880	*A. thaliana*	3.00E-20	92%	BAH19602.1		0
23_4	208	MAP kinase	*A. thaliana*	1.00E-20	98%	CAB63149.1	0	
139_4	300	Putative leucine-rich repeat receptor-like protein kinase	*A. thaliana*	4.00E-25	55%	NP_200956.1	0	
72_1	238	CBL-interacting protein kinase 19	*Populus trichocarpa*	8.7	89%	ABJ91226.1	0	
39_3	200	At1g25390/F2J7_14	*A. thaliana*	3.00E-23	81%	AAK97715.1	0	
12_2	250	CDK activating kinase	*Nicotiana tabacum*	3.7	46%	BAF75824.1	+	
22_2	252	Serine/threonine protein kinase ATR	*M. truncatula*	6.00E-30	83%	XP_003592675.1	+	
235_3	285	Receptor-like protein kinase	*M. truncatula*	9.00E-11	57%	XP_003621121.1	+	
110_1	408	Receptor-like protein kinase	*A. thaliana*	2.00E-31	55%	BAA96958.1		0
99_1	342	Protein phosphatase 2C (PP2C)	*Fagus sylvatica*	6.00E-30	71%	CAB90633.1	2.6	3.7
99_2	273	C3H4 type zinc finger protein	*A. thaliana*	7.00E-28	64%	NP_194986.2	+	
54_1	318	AT5g57360/MSF19_2	*A. thaliana*	1.00E-45	75%	AAK64006.1	+	
57_1	246	E3 ligase SAP5	*A. thaliana*	2.00E-37	84%	NP_566429.1	+	
234_1	306	Root phototropism protein 2	*A. thaliana*	9.00E-29	60%	NP_001031446.1	2.8	3.4
96_1	229	E3 ubiquitin-protein ligase BRE1-like protein	*M. truncatula*	2.8	29%	XP_003637493.1	0	
187_1	314	Skp1-like protein 1	*Prunus avium*	4.00E-51	85%	AFJ21662.1	0	
120_2	227	Polyubiquitin	*Cicer arietinum*	8.00E-39	100%	BAA76429.1	0.1	
158_2	313	Putative E3 ubiquitin-protein ligase XBAT31 isoform 2	*Vitis vinifera*	2.00E-18	63%	XP_002283974.1		+
73_1	327	F-box family protein	*Citrus trifoliata*	4.00E-64	98%	ACL51019.1		0
112_1	202	F-box with WD-40 2	*A. thaliana*	1.00E-04	81%	NP_567343.1		0
38_3	212	Drought-inducible cysteine proteinase RD19A precursor	*A. thaliana*	1.00E-15	86%	BAD94010.1	6.0	0.3
81_1	234	Metalloendopeptidase/zinc ion binding protein	*A. thaliana*	1.00E-31	84%	NP_568608.2	+	
38_4	261	Serine carboxypeptidase II-3	*M. truncatula*	7.00E-21	74%	XP_003589243.1	5.9	
73_4	143	Proteasome component (PCI) domain protein	*A. thaliana*	2.00E-07	69%	NP_850994.1		+
240_1	359	RHOMBOID-like protein 3	*A. thaliana*	8.00E-38	65%	NP_196342.1		+
39_1	248	Clp protease proteolytic subunit	*Citrus sinensis*	2.00E-29	100%	YP_740501.1	0	
145_1	319	Subtilase family protein	*A. thaliana*	3.00E-32	62%	NP_199378.1		0
67_1	315	Aminopeptidase family protein	*A. thaliana*	2.00E-45	85%	NP_179997.1		0
75_1	251	Papain family cysteine protease	*A. thaliana*	3.00E-26	85%	NP_567489.1		0
138_4	320	AT4G01850	*A. thaliana*	3.00E-59	93%	BAH20274.1		+
245_1	270	Methionine synthase	*Carica papaya*	2.00E-45	98%	ABS01352.1	0	
231_4	216	N-carbamoylputrescine amidase	*A. thaliana*	6.00E-10	76%	NP_565650.1	0.1	
61_2	289	2-oxoglutarate-dependent dioxygenase	*P. trichocarpa*	1.00E-07	74%	XP_002313083.1	+	
251_3	276	Cystathionine beta-synthase domain-containing protein	*A. thaliana*	8.00E-45	89%	NP_195409.1		0
***Stress responses***								
118_1	207	Inorganic pyrophosphatase 1	*A. thaliana*	2.00E-16	83%	NP_565052.1	0	3.3
148_2	317	Nudix hydrolase 19	*A. thaliana*	2.00E-48	78%	NP_197507.1	0	+
59_1	346	Fe (II)/ascorbate oxidase family protein SRG1	*A. thaliana*	2.00E-16	71%	NP_173145.1		0
137_2	156	Thioredoxin superfamily protein	*A. thaliana*	3.00E-10	58%	NP_198706.1	+	
68_3	146	Thioredoxin superfamily protein	*A. thaliana*	3.00E-07	59%	NP_201385.2	0.1	
2_1	276	Group 5 late embryogenesis abundant protein (LEA5)	*Citrus unshiu*	1.00E-35	94%	ABD93882.1	3.0	
125_1	389	Thaumatin-like protein 1	Apple tree	9.00E-48	69%	JC7201	+	
99_5	190	Protein sodium-and lithium-tolerant 1	*A. thaliana*	1.00E-23	92%	NP_973625.1	0	
104_3	171	Transducin/WD40 domain-containing protein (AtATG18a, AT3G62770)	*A. thaliana*	3.00E-20	94%	NP_001030918.4		0
109_1	257	Cold regulated 314 thylakoid membrane 2	*A. thaliana*	1.00E-19	56%	NP_564327.1		0
150_2	238	Universal stress protein A-like protein	*M. truncatula*	4.00E-27	71%	XP_003591417.1	0.2	
***Signal transduction***								
182_2	117	Signal recognition particle 54 kDa protein 2	*Solanum lycopersicum*	7.00E-07	93%	NP_001234428.1	0	
108_2	257	14-3-3 protein	*Dimocarpus longan*	6.00E-38	93%	ACK76233.1	0	
200_1	240	Heterotrimeric GTP-binding protein subunit beta 1	*Nicotiana tabacum*	3.00E-39	94%	AAG12330.1	0	
70_2	252	Pseudo-response regulator 5	*Castanea sativa*	5.00E-12	86%	ABV53464.1		+
***Cell transport***								
26_1	342	H^+^-ATPase 6, plasma membrane-type	*A. thaliana*	1.00E-38	97%	NP_178762.1	+	
124_3	166	Calcium-transporting ATPase 1, endoplasmic reticulum-type (ECA1)	*A. thaliana*	2.00E-14	83%	NP_172259.1	3.1	
66_1	177	Heavy metal ATPase	*P. trichocarpa*	4.00E-15	78%	XP_002303580.1	+	
97_1	201	Proton pump-interactor 1 (PPI1, AT4G27500)	*A. thaliana*	3.00E-12	56%	BAH19433.1	+	
53_1	340	ABC transporter G family member 40	*A. thaliana*	9.00E-35	67%	NP_173005.1	+	
210_1	247	Copper transporter	*P. trichocarpa*	2.00E-15	64%	XP_002298334.1	+	
178_1	297	Cyclic nucleotide-gated ion channel 1	*A. thaliana*	0.002	50%	NP_200125.1	+	
49_3	252	Vacuolar-sorting receptor 3	*A. thaliana*	1.00E-40	77%	NP_179081.1	+	
137_1	249	Vacuolar protein-sorting-associated protein 37-1	*A. thaliana*	0.48	63%	NP_190880.1	+	
63_1	357	Vesicle-associated membrane protein-associated protein	*M. truncatula*	3.00E-05	70%	XP_003608721.1	+	
51_1	316	SecY protein transport family protein	*A. thaliana*	2.00E-51	87%	NP_174225.2	+	
250_2	263	Fat-free-like protein	*M. truncatula*	1.00E-32	82%	XP_003591407.1	+	
79_2	237	Non-specific lipid-transfer protein	*M. truncatula*	1.00E-04	53%	XP_003610781.1	2.5	
67_3	268	Sieve element occlusion protein 1	*Nicotiana tabacum*	6.00E-23	65%	AFN06072.1	+	+
89_2	230	AT5g24810/F6A4_20	*A. thaliana*	1.00E-04	75%	AAK82520.1	0	
6_1	368	Protein transport protein SEC61 gamma subunit	*Zea mays*	2.00E-04	92%	NP_001150911.1	0	
249_2	370	Putative beta-subunit of adaptor protein complex 3, PAT2	*A. thaliana*	2.00E-15	42%	NP_567022.1	0	0
61_1	228	Sugar transporter ERD6-like 5	*A. thaliana*	7.00E-15	57%	NP_564665.3		0
179_2	225	Metal tolerance protein	*P. trichocarpa*	6.00E-26	70%	XP_002312066.1		0
51_4	221	Kinesin-related protein	*M. truncatula*	0.38	35%	XP_003612133.1		+
36_2	319	Bidirectional sugar transporter SWEET7	*A. thaliana*	5.00E-08	60%	NP_567366.1		+
***Cell wall and cytoskeleton modification***								
49_4	210	Caffeic acid 3-O-methyltransferase	*M. truncatula*	9.00E-23	68%	XP_003602597.1		0
125_2	145	Caffeic acid O-methyltransferase 3	*Gossypium hirsutum*	2.00E-05	55%	ACZ06242.1	0.2	
10_3	274	Chitinase	*Citrus sinensis*	3.00E-54	94%	CAA93847.1	0	0
249_4	217	Cellulose synthase	*Populus tremuloides*	1.00E-20	83%	AAO25581.1	0.2	
33_3	249	O-methyltransferase 1	*A. thaliana*	1.00E-33	74%	AAB96879.1	+	
241_1	326	LIM domain-containing protein	*A. thaliana*	1.00E-64	94%	NP_195404.6	+	
124_2	385	UDP-glucose flavonoid 7-O-glucosyltransferase	*M. truncatula*	4.00E-12	73%	XP_003629628.1	+	
3_3	225	UDP-glucosyltransferase family 1 protein	*Citrus sinensis*	6.00E-36	96%	ACS87993.1	+	
70_4	176	Limonoid UDP-glucosyltransferase	*Citrus sinensis*	2.00E-26	98%	ACD14147.1	+	
63_2	228	Putative glucosyltransferase	*A. thaliana*	2.00E-20	63%	AAM61749.1	3.9	
***Other and unknown processes***								
229_4	181	Phytoene synthase	*Citrus unshiu*	1.00E-26	95%	AAF33237.1	0	
231_1	316	Strictosidine synthase family protein	*A. thaliana*	2.00E-28	68%	NP_191262.2	0.4	2.6
72_3	194	Calcium-dependent lipid-binding domain-containing protein	*A. thaliana*	8.00E-19	78%	NP_564576.1	+	+
135_2	335	Oxidoreductase family protein	*Arabidopsis lyrata subsp.lyrata*	3.00E-40	65%	XP_002874584.1		0
5_2	262	Alkaline-phosphatase-like protein	*A. thaliana*	7.00E-44	89%	NP_194697.1	0	
10_5	147	Protein tolB	*M. truncatula*	2.00E-06	55%	XP_003630471.1	+	
231_2	285	Cofactor of nitrate reductase and xanthine dehydrogenase 3	*A. thaliana*	5.00E-35	83%	NP_171636.1		3.9
51_3	256	Neutral/alkaline non-lysosomal ceramidase	*A. thaliana*	3.00E-14	71%	NP_172218.1		0.5
229_2	207	PQ-loop repeat family protein	*A. lyrata subsp. lyrata*	2.00E-22	74%	XP_002870687.1	+	
71_4	206	Metallo-beta-lactamase domain-containing protein	*A. thaliana*	9.00E-19	66%	NP_564334.1	+	
117_3	214	Oligosaccharyltransferase complex/magnesium transporter family protein	*A. thaliana*	5.00E-17	60%	NP_176372.1	0	
146_3	337	Mitochondrial protein, putative	*M. truncatula*	1.00E-24	74%	XP_003588355.1	0.4	0.3
20_1	287	AT1G16560	*A. thaliana*	2.00E-42	74%	BAH19866.1	+	
117_1	338	At2g27385	*A. lyrata subsp. lyrata*	8.00E-15	91%	XP_002880912.1	0.2	
173_1	290	SOUL heme-binding protein	*A. thaliana*	1.00E-40	90%	NP_197514.2	0	
122_1	166	AT-LS1 product	*A. thaliana*	2.00E-21	86%	CAA41632.1	0	
77_2	231	Alpha/beta-hydrolase family protein	*A. thaliana*	3.00E-36	94%	NP_196943.1	1.8	
99_3	265	Conserved hypothetical protein	*Ricinus communis*	0.069	44%	XP_002511001.1	0	+
229_1	271	Conserved hypothetical protein	*R. communis*	2.00E-09	90%	XP_002532497.1	0	
70_1	267	Predicted protein	*Micromonas pusilla CCMP1545*	3.00E-49	94%	XP_003064993.1	+	+
123_1	364	PREDICTED: exportin-4-like	*Vitis vinifera*	9.00E-47	81%	XP_002266608.2	+	
23_1	308	Predicted protein	*P. trichocarpa*	0.062	34%	XP_002317402.1	+	
232_2	246	Predicted protein	*P. trichocarpa*	5.00E-12	48%	XP_002319603.1	0.1	
237_1	265	PREDICTED: uncharacterized protein LOC100776190	*Glycine max*	5.8	36%	XP_003524378.1	0	
242_1	245	PREDICTED: uncharacterized protein LOC100789831	*G. max*	2.00E-07	60%	XP_003520084.1	+	
69_2	244	PREDICTED: uncharacterized protein LOC100853355	*Vitis vinifera*	0.008	49%	XP_003634177.1	0	
130_2	210	Uncharacterized protein	*A. thaliana*	6.00E-21	79%	NP_176682.1		1.7
252_1	301	Uncharacterized protein	*A. thaliana*	8.00E-16	56%	NP_001031080.1	0	
97_2	163	Unnamed protein product	*Vitis vinifera*	0.079	42%	CBI21631.3	0	7.0
91_2	270	Hypothetical protein	*A. thaliana*	0.19	54%	AAD21766.1		3.9
9_1	255	Hypothetical protein MTR_5g051130	*M. truncatula*	1.00E-11	100%	XP_003614394.1	+	

Expression ratio: 0 means TDFs were only detected in control leaves; + means TDF were only detected in the B-toxic leaves. #: Number; BT: B-toxicity; CK: Control; CG: *C. grandis*; CS: *C. sinensis*. Functional classification was performed based on the information reported for each sequence by The Gene Ontology (http://amigo1.geneontology.org/cgi-bin/amigo/blast.cgi) and Uniprot (http://www.uniprot.org/). Relative expression ratio was obtained by gel image analysis, which was performed with PDQuest version 8.0.1 (Bio-Rad, Hercules, CA, USA).

### Validation of cDNA-AFLP data using qRT-PCR

**Figure 5:**
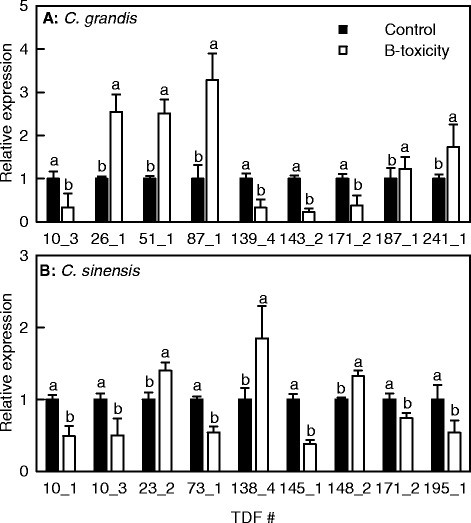
**Effects of B**-**toxicity on gene expression of** ***Citrus grandis*** **(A)** **and** ***Citrus sinensis*** **(B)** **leaves.** **(A)** Relative expression levels of genes encoding chitinase (TDF #10_3), H^+^-ATPase 6 (TDF #26-1), secY protein transport family protein (TDF #51_1), pyruvate dehydrogenase E1 component subunit β (TDF #87-1), putative leucine-rich repeat receptor-like protein kinase (TDF #139_4), Rubisco small subunit precursor (TDF #143-2), PORC (TDF #171_2), Skp1-like protein 1 (TDF #187_1) and LIM domain-containing protein (TDF #241_1). **(B)** Relative expression levels of genes encoding fatty acid hydroperoxide lyase (TDF #10_1), chitinase (TDF #10_3), glyceraldehyde-3-phosphate dehydrogenase B (TDF #23-2), F-box family protein (TDF #73-1), AT4G01850 (TDF #138_4), subtilase family protein (TDF #145_1), Nudix hydrolase 19 (TDF #148_2), PORC (TDF #171_2) and sugar-dependent1 (TDF #195_1). Bars represent means ± SE (n =3). Different letters above the bars indicate a significant difference at *P* <0.05.

In this study, nine TDFs from *C. sinensis* leaves and nine TDFs from *C. grandis* ones were selected for qRT-PCR analysis in order to validate their expression patterns obtained by cDNA-AFLP analysis. Except for two TDFs (i.e., TDFs #187_1 and 195_1), the expression profiles of all the TDFs obtained by qRT-PCR were in agreement with the expression patterns produced by cDNA-AFLP (Figure 5). This technique was thus validated in 88.9% of cases. In addition to gene family complexity, the changes in the intensity of individual bands in the cDNA-AFLP gels might be responsible for the discrepancies between qRT-PCR and cDNA-AFLP analysis.

## Discussion

### *C. sinensis* displayed higher B-tolerance than *C. grandis*

Our results showed that the effects of B-toxicity on plant growth (Figure 1A-C), and leaf gas exchange, pigments, total soluble protein, MDA (Figure 3) and P (Figure 2C) were more pronounced in *C. grandis* than in *C. sinensis* seedlings, meaning that *C. sinensis* has higher B-tolerance than *C. grandis*. The present work, like that of the previous workers [8,13,15], indicates that the major of B in B-toxic citrus plants was accumulated in the leaves (Figure 2A and B). As shown in Figure 2B, B concentration was not lower in *C. sinensis* than in *C. grandis* leaves regardless of B concentration in the nutrient solution, indicating that *C. sinensis* leaves may tolerate higher level of B. Similar result has been obtained by Huang et al. [13]. Here we isolated 67 up-regulated and 65 down-regulated TDFs from B-toxic *C. grandis* leaves, whilst only 31 up-regulated and 37 down-regulated TDFs from B-toxic *C. sinensis* ones (Figure 4), suggesting that B-toxicity affects *C. sinensis* leaves gene expression less than *C. grandis* ones. These data also support above inference that *C. sinensis* leaves may tolerate higher level of B.

We found that CO_2_ assimilation was lower in toxic leaves than in control leaves, while stomatal conductance was not lower in the former (Figure 3A-C), implies that B-toxicity-induced inhibition of CO_2_ assimilation in two citrus species is primarily due to non-stomatal factors. Similar results have been obtained on B-toxic *C. grandis* and *C. sinensis* [13,14], ‘Navelina’ orange and ‘Clementine’ mandarin plants grafted on sour orange and Swingle citrumelo rootstocks [11,12], Newhall and Skagg’s Bonanza navel orange plants grafted on Carrizo citrange and trifoliate orange [9].

### Leaf carbohydrate and energy metabolism

Since B-toxicity decreased CO_2_ assimilation (Figure 3A), genes involved in photosynthesis and related biological processes might be affected by B-toxicity. As expected, 16 TDFs in *C. grandis* leaves and 14 TDFs in *C. sinensis* ones related to carbohydrate and energy metabolism were altered under B-toxicity (Table 2 and Figure 4). We found that B-toxicity decreased the transcript level of ribulose-1,5-bisphosphate (RuBP) carboxylase/oxygenase (Rubisco) small subunit precursor (TDF #143_2) gene in *C. grandis* leaves (Table 2), which agrees with the previous report that B-toxicity decreased the activity of Rubisco in *C. grandis* leaves [14]. Hudson et al. showed that the reduction of Rubisco concentration by anti-small subunit led to decreased photosynthesis in transgenic tobacco plants, but unchanged stomatal conductance [16]. Also, the mRNA abundances of photosystem II (PSII) 32 kDa protein (PsbA, TDF #251_1), chloroplast PSII oxygen-evolving complex 23 kDa polypeptide (TDF #112_2) and NifU-like protein (TDF #239_4) genes were down-regulated in B-toxic *C. grandis* leaves (Table 2). Khan et al. reported that *PsbA* knockout tobacco plants lacked PSII activity, accompanied by promoted senescence [17]. By using differential RNA interference (RNAi), Ishihara et al. demonstrated that PSII activity was linearly correlated with the total amount of PsbP (PSII 23 kDa protein) [18]. Ifuku et al. reported that PsbP is essential for the regulation and stabilization of PSII in higher plants [19]. Yabe et al. proposed that *Arabidopsis* chloroplastic NifU-like protein, which can act as a Fe-S cluster scaffold protein, was required for biogenesis of ferredoxin and photosystem I (PSI) [20]. B-toxicity-induced decreases in the transcript levels of PsbA, chloroplast PSII oxygen-evolving complex 23 kDa polypeptide and NifU-like protein genes agree with our report that B-toxicity impaired the whole photosynthetic electron transport from PSII donor side up to the reduction of end acceptors of PSI in *C. grandis* leaves [14]. By contrast, B-toxicity increased the transcript levels of chloroplast PSII oxygen-evolving complex 23 kDa polypeptide (TDF #112_2) and glyceraldehyde-3-phosphate dehydrogenase B (TDE #23_2) in *C. sinensis* leaves (Table 2). NADP-glyceraldehyde-3-phosphate dehydrogenase is one of the two chloroplast enzymes which catalyze the reduction of 3-phosphoglycerate to triose phosphate [21]. However, the expression of Rubisco activase (TDF #6_4) gene in *C. sinensis* leaves decreased in response to B-toxicity (Table 2). Generally speaking, B-toxic *C. sinensis* leaves had higher expression levels of photosynthetic genes than B-toxic *C. grandis* ones. This might be responsible for the greater decrease in CO_2_ assimilation in B-toxic *C. grandis* leaves compared with B-toxic *C. sinensis* ones. It is noteworthy that the mRNA level of gene encoding sedoheptulose-1,7-bisphosphatase (SBPase, TDF #249_3), a key factor for the RuBP regeneration, was up-regulated in B-toxic leaves of the two citrus species (Table 2). Harrison et al. showed that a small decrease in SBPase activity caused a decline in CO_2_ assimilation by reducing the capacity for RuBP regeneration [22]. Lefebvre et al. observed that transgenic tobacco plants over-expressing *SBPase* had enhanced photosynthesis and growth from an early stage in development [23]. Wang reported that transgenic tomato plants over-expressing *SBPase* were more tolerance to low temperature and had higher photosynthetic capacity under low temperature [24]. Therefore, the up-regulation of *SBPase* might be an adaptive response to B-toxicity.

As shown in Table 2, B-toxicity decreased leaf expression levels of three genes [i.e., ADP-glucose pyrophosphorylase (TDF #235_2) in *C. sinensis*, starch branching enzyme I (TDF #42_1) in *C. grandis* and glucose-1-phosphate adenylyltransferase large subunit 1 (TDF #59_2) in the two citrus species] related to starch biosynthesis, which agrees with the previous report that B-toxicity decreased starch concentration in *C. grandis* leaves [14].

B-toxicity increased the mRNA levels of three genes encoding citrate synthase (TDF #75-2), pyruvate dehydrogenase E1 component subunit beta (TDF #87_1) and aconitate hydratase 3 (TDF #33-2) in *C. grandis* leaves (Table 2), indicating that tricarboxylic acid cycle might be up-regulated in B-toxic *C. grandis* leaves. Similarly, the transcript level of a glycolysis gene encoding 2,3-bisphosphoglycerate-independent phosphoglycerate mutase (TDF #161_3) was enhanced in B-toxic *C. sinensis* leaves (Table 2). However, the mRNA levels of plastidial pyruvate kinase 3 (TDF #35_1) and aconitate hydratase 1 (TDF #33_2) genes were reduced in B-toxic *C. sinensis* leaves (Table 2). There is evidence showing that plastidic pyruvate kinase plays a key role in fatty acid synthesis by controlling the supply of ATP and pyruvate for *de novo* fatty acid synthesis in plastids [25]. Thus, the fatty acid metabolism in B-toxic *C. sinensis* leaves might be impaired due to decreased plastidic pyruvate kinase.

In *Arabidopsis*, three NADPH: protochlorophyllide oxidoreductases (PORs), denoted as PORA, PORB, and PORC participate in mediating the light-dependent protochlorophyllide reduction [26]. Pattanayak and Tripathy showed that over-expression of *PORC* in *Arabidopsis* led to coordinated up-regulation of gene/protein expression of several Chl biosynthetic pathway enzymes, thus enhancing Chl synthesis, and that the ^1^O_2_-mediated photo-oxidative damage in transgenic plants overexpressing *PORC* was minimal under high light stress [27]. The observed lower transcript level of *PORC* (TDF #171_2) in B-toxic *C. grandis* and *C. sinensis* leaves (Table 2) agrees with the results that B-toxicity decreased the concentration of Chl a + b in citrus leaves (Figure 3E).

Cytochrome P450s play a key role in biotic and abiotic stresses. Transgenic tobacco and potato plants expressing *cytochrome P450* with increased monooxygenase activity tolerated better oxidative stress after herbicide treatment [28]. We found that B-toxicity increased the expression levels of genes encoding cytochrome P450 (TDF #5_1) and cytochrome P450 like protein (TDF #76-1) in *C. grandis* leaves (Table 2), which agrees with the previous report that some of the 49 cytochrome P450 genes in *Arabidopsis* were upregulated by biotic (i.e., *Alternaria brassicicola* and *Alternaria alternata*) and abiotic [i.e., drought, high salinity, low temperature, hormones, paraquat, rose bengal, UV stress (UV-C), mechanical wounding and heavy metal stress (CuSO_4_)] stresses [29]. Thus, the up-regulation of *cytochrome P450s* in B-toxic *C. grandis* leaves might be an adaptive response. However, B-toxicity decreased the expression of *cytochrome P450* in *Arabidopsis* roots [7].

Taken all together, we isolated eight up-regulated and eight down-regulated TDFs from B-toxic *C. grandis* leaves, and five up-regulated and nine down-regulated from B-toxic *C. sinesnsis* ones. Among these differentially expressed TDFs, only *SBPase* (TDF #249_3) and *PORC* (TDF #171_2) were similarly affected by B-toxicity in the two species (Table 2). These results demonstrated that the transcript profiles in the two species were differentially altered under B-toxicity.

### Leaf lipid metabolism

Allene oxide synthase (AOS) and hydroperoxide lyase (HPL) branches of the oxylipin pathway, which are responsible for the production of jasmonates and aldehydes, respectively, participate in a range of stresses. Recently, Liu et al. showed that depletion of rice *OsHPL3* greatly stimulated the jasmonic acid-governed defense response [30]. Therefore, the AOS pathway and jasmonate level might be up-regulated in the B-toxic *C. sinensis* leaves due to decreased expression of *fatty acid HPL* (TDF #10_1; Table 2), thus contributing to B-tolerance. In addition, B-toxicity also affected the transcript levels of three genes [i.e., *plastidial pyruvate kinase 3* (TDF #35_1), *sugar*-*dependent1* (TDF #195_1) and *3*-*oxoacyl*-*reductase* (TDF #233_3)] related to lipid metabolism in *C. sinensis* leaves (Table 2). Thus, lipid metabolism might be altered in B-toxic *C. sinensis* leaves.

Tang et al. reported that transgenic tobacco plants over-expressing *acyl carrier protein* (*ACP*)-*1* (or expressing antisense *ACP1*) exhibited an increase (or decrease) in leaf concentrations of total lipids and the main fatty acids, and were more tolerant (or sensitive) to cold stress [31]. Branen et al. showed that reduction of *ACP4* by antisense RNA led to a decrease in total leaf lipids and decreased photosynthetic efficiency, and concluded that *ACP4* might play a major role in the biosynthesis of fatty acids for chloroplast membrane development [32]. The lower transcript level of gene encoding ACP1, chloroplastic-like (TDF #8_1) in B-toxic *C. grandis* leaves (Table 2) means that fatty acid biosynthesis in these leaves might be impaired. However, the expression of α/β-hydrolase domain-containing protein (TDF #194_1) and phospholipase-like protein (PEARLI 4) domain-containing protein (TDF #186_4) genes were up-regulated in B-toxic *C. grandis* leaves (Table 2).

### Leaf nucleic acid metabolism

As shown in Table 2, eight up-regulated genes (TDFs #52_1, 49_1, 72_4, 120_1, 44_1, 159_2, 164_1 and 73_2) and nine down-regulated genes (TDFs #250_3, 157_2, 11_1, 71_3, 67_4, 10_4, 22_3, 104_1 and 68_2) were isolated from B-toxic *C. grandis* leaves, while only five up-regulated genes (TDFs #73_2, 250_3, 157_2, 60_1 and 131_1) and four down-regulated genes (TDFs #164_1, 71_3, 186_1 and 108_1) were identified in B-toxic *C. sinensis* leaves. Obviously, B-toxicity affected nucleic acid metabolism more in *C. grandis* leaves than in *C. sinensis* ones. This agrees with our inference that *C. sinensis* may tolerate higher level of B.

### Leaf protein and amino acid metabolism

All these differentially expressed TDFs encoding chloroplatic translation initiation factor IF-2 (TDF #236_1) involved in promoting the binding of formylmethionyl-tRNA to 30 S ribosomal subunits, eukaryotic release factor 1–3 (TDF #117_4) involved in the termination step of protein synthesis, EMB1241 (At5g17710; TDF #93_3 ) related to protein folding and stabilization, Ankyrin repeat domain-containing protein (TDF #73_3) involved mainly in mediating protein-protein interactions, and ribosomal proteins [i.e., 50S ribosomal protein L15 (TDF #179_4), 30S ribosomal protein S17 (TDF #105_1), putative 60S ribosomal protein L6 (TDF #99_6) and 60S ribosomal protein L4_1 (TDF #186_2) ] related to mature ribosome assembly and translation processes except for SHEPHERD (TDF #93_2) involved in the correct folding and/or complex formation of CLAVATA (CLV) proteins [33], 60S ribosomal protein L23 (TDF #129_2) and 60S ribosomal protein L10B (TDF #161_1), were down-regulated in B-toxic *C. grandis* leaves (Table 2), indicating that B-toxicity impairs protein biosynthesis in *C. grandis* leaves [34,35]. By contrast, only three down-regulated genes [30S ribosomal protein S17 (TDF #105_1), chaperonin 20 (TDF #98_1) involved in protein folding and stabilization and AT5G47880 (TDF #69_3) involved in the termination step of protein synthesis] were detected in B-toxic *C. sinensis* leaves (Table 2). These results demonstrated that B-toxicity affected protein biosynthesis more in the former than in the latter. This agrees with our data that B-toxicity only decreased total soluble protein concentration in *C. grandis* leaves (Figure 3H).

Here we observed four down-regulated genes [i.e., mitogen-activated protein (MAP) kinase (TDF #23_4), putative leucine-rich repeat receptor-like protein kinase (TDF #139-4), CBL-interacting protein kinase 19 (TDF #72_1) and At1g25390/F2J7_14 (TDF #39_3)] and three up-regulated genes [i.e., CDK activating kinase (TDF #12_2), serine/threonine protein kinase ATR (TDF #22_2) and receptor-like protein kinase (TDF #235_3) ] involved in phosphorylation and one up-regulated gene [i.e., protein phosphatase 2C (TDF #99_1)] involved in dephosphorylation in B-toxic *C. grandis* leaves, while only one down-regulated gene [i.e., receptor-like protein kinase (TDF #110_1)] and one up-regulated gene [i.e., protein phosphatase 2C (TDF #99_1)] in B-toxic *C. sinensis* leaves (Table 2). This means that *C. sinensis* leaves might achieve a better balance between phosphorylation and dephosphorylation than *C. grandis* ones under B-toxicity, which might contribute to the B-tolerance of *C. sinensis*.

Inactive (i.e., incorrect folding) and futile proteins for cell are tagged by ubiquitin for proteolysis [36]. In this study, we found four up-regulated genes [i.e., C3H4 type zinc finger protein (TDF #99_2), AT5g57360/MSF19_2 (TDF #54_1), E3 ligase SAP5 (TDF #57_1) and root phototropism protein 2 (TDF #234_1)] and three down-regulated genes [i.e., E3 ubiquitin-protein ligase BRE1-like protein (TDF #96_1), Skp1-like protein 1 (TDF #187_1) and polyubiquitin (TDF #120_2)] involved in ubiquitination in B-toxic *C. grandis* leaves, and one up-regulated gene [i.e., putative E3 ubiquitin-protein ligase XBAT31 isoform 2 (TDF #158_2)] and two down-regulated genes [i.e., F-box family protein (TDF #73_1) and F-box with WD-40 2 (TDF #112_1)] involved in ubiquitination in B-toxici *C. sinensis* leaves. This indicates that ubiquitination might be involved in the adaptive response of citrus leaves to B-toxicity. Plant proteases has been shown to play key roles in controlling strict protein quality and degrading specific sets of proteins in response to environmental stresses [37]. As expected, several genes (TDFs #38_3, 81_1, 38_4, 73_4, 240_1, 39_1, 145_1, 67_1 and 75_1) involved in proteolysis were altered in B-toxic *C. grandis* and *C. sinensis* leaves (Table 2).

S-adenosylmethionine (AdoMet) participates in a number of essential metabolic pathways in plants and is the principal biological methyl donor. AdoMet-dependent methylation is essential for keeping cellular functions in plants [38]. Methionine synthase, which catalyzes the last reaction in *de novo* methionine synthesis, also serves to regenerate the methyl group of AdoMet. As shown in Table 2, B-toxicity increased the expression of AT4G01850 (TDF #138_4) involved in AdoMet biosynthesis in *C. sinensis* leaves, but decreased *Methionine synthase* expression (TDF #245_1) in *C. grandis* leaves, which might contribute to the higher tolerance of *C. sinensis* leaves to B-toxicity than that of *C. grandis* ones.

N-carbamoylputrescine amidase (TDF #213_4) involved in polyamine (putrescine) biosynthesis were down-regulated in B-toxic *C. grandis* leaves (Table 2). This means that the biosynthesis of polyamine might be inhibited in B-toxic *C. grandis* leaves, which disagrees with the previous report that 1000 μM B increased leaf concentration of putrescine in B-sensitive barley cultivar, but decreased its concentration in B-tolerant one [39].

The up-regulation of 2-oxoglutarate-dependent dioxygenase gene (TDF #61_2) in B-toxic *C. grandis* leaves (Table 2) agrees with the reports that B-toxicity stimulated the general amino acid control system in *Saccharomyces cerevisiae* [35] and that the concentration of total amino acids in tomato leaves increased under B-toxicity [40]. Evidence shows that 2-oxoglutarate-dependent dioxygenase participates in glucosinolate biosynthesis [41]. Thus, the concentration of glucosinolates might be enhanced in B-toxic *C. grandis* leaves.

There is evidence showing that a few cystathionine-β-synthase (CBS) domain-containing proteins (CDCPs) play a role in plant stress response/tolerance and development [42]. Overexpression of *OsCBSX4* improved tobacco plant tolerance to salinity, oxidative, and heavy metal stresses [43]. We observed that B-toxicity decreased the transcript level of *CDCP* (TDF #251_3) in *C. sinensis* leaves (Table 2), as obtained on manganese (Mn)-toxic *C. grandis* leaves [44]. However, B-deficient *C. sinensis* roots had higher level of CBS family protein [45]. Singh et al. observed that the expression of *OsCBSX4* was up-regulated under high salinity, heavy metal, and oxidative stresses at seedling stage of a salt tolerant (Pokkali) rice cultivar, whilst its expression was upregulated only under NaCl stress, downregulated under heavy metal stress and kept unchanged under oxidative stress in a salt sensitive (IR64) rice one [43]. Taken all together, the influence of stresses on expression of CDCP genes deponds on the kinds of stresses and plant species/cultivars.

### Leaf stress responses

Inorganic pyrophosphatase (PPase), which cleaves pyrophosphate molecules to liberate two molecules of inorganic phosphate, are essential for the viability of organisms, because the removal of pyrophosphate, a by-product of a host of biosynthetic reactions, is required for preventing the inhibition of thermodynamically unfavorable reactions [46,47]. George et al. observed that *Nicotiana benthamiana* plants lacking plastidial soluble PPase exhibited reduced drought tolerance as a result of the impaired leaf anabolic pathways [46]. The up-regulation of *PPase 1* (TDF #118_1) in B-toxic *C. sinensis* leaves (Table 2) might be an adaptive response to B-toxicity. By contrast, its expression (TDF #118_1) was down-regulated in B-toxic *C. grandis* leaves (Table 2).

Because leaf CO_2_ assimilation was decreased in B-toxic leaves (Figure 3A), less of the absorbed light energy was utilized in photosynthetic electron transport in these leaves, particularly under high light. Thus, reactive oxygen species (ROS) production might be enhanced in B-toxic leaves because of more excess absorbed photon flux [14]. In addition to various ROS scavenger enzymes, “house-keeping” enzymes such as Nudix hydrolases (NUDXs) also play a role in ROS scavenging. Ogawa et al. [48] and Ishikawa et al. [49] showed that transgenic *Arabidopsis* plants overexpressing *AtNUDX2* and *AtNUDX7* exhibited higher tolerance to oxidative stress than wild type plants. Therefore, the higher expression level of *NUDX19* (TDF #148_2) in B-toxic *C. sinensis* leaves might be an adaptive response to B-toxicity (Table 2). However, its expression level (TDF #148_2) in *C. grandis* leaves decreased in response to B-toxicity (Table 2).

Up to 10% of the ascorbate content of the whole leaf is localized in the apoplast, where it forms the first line of defense against external oxidants [50]. In the apoplast, ascorbate oxidase (AO) oxidizes ascorbate to the unstable radical monodehydroascorbate which rapidly disproportionates to yield dehydroascorbate and ascorbate, thus participating in the regulation of the redox state of ascorbic acid pool. AO has been suggested to play a role in cell expansion *via* the modulation of redox control of the apoplast [51]. Pignocchi et al. [52] showed that enhanced AO activity decreased the concentration and the redox state of ascorbic acid pool in the apoplast, whereas reduced AO activity increased its amount and redox state in the apoplast. Overexpression of *AO* in the apoplast of tobacco resulted in lowered capacity for scavenging ROS in the leaf apoplast accompanied by increased sensitivity to ozone [53]. Fotopoulos et al. [50] observed that *AO*-overexpressing transgenic tobacco plants had increased sensitivity to various oxidative stress-promoting agents accompanied by a general suppression of the plant antioxidative metabolism. By contrast, a diminution in AO activity improved tomato yield under water deficit [54]. The down-regulation of gene encoding Fe (II)/ascorbate oxidase family protein SRG1 (TDF #59_1) in B-toxic *C. sinensis* leaves (Table 2) might increase the amount and the redox state of AA pool in the apoplast, thus enhancing the B-tolerance.

Thioredoxins, which participates in supplying reducing power to reductases required for detoxifying lipid hydroperoxides or repairing oxidized proteins, play key roles in plant tolerance of oxidative stress [55]. We found that the expression level of *thioredoxin superfamily protein* (TDF #137_2) was up-regulated in B-toxic *C. grandis* leaves (Table 2), indicating that thioredoxins might be involved in the ROS detoxification. However, the transcript level of thioredoxin superfamily protein (TDF #68_3) gene was down-regulated in B-toxic *C. grandis* leaves.

Our finding that B-toxicity increased the expression level of *group 5 late embryogenesis abundant protein* (LEA5, TDF #2_1) in *C. grandis* leaves (Table 2) agrees with the previous report that drought, heat and salt stresses stimulated the expression of LEA5 in citrus leaves [56]. Accumulation of AtRAB28 (LEA5) protein in *Arabidopsis* through transgenic approach improved the germination rate under standard conditions or salt and osmotic stresses and the cation toxicity tolerance [57]. Also, B-toxicity increased the transcript level of *thaumatin*-*like protein 1* (TLP1, TDF #125_1) in *C. grandis* leaves (Table 2). The family of thaumatin-like proteins (also designated PR-5), which comprises proteins with various functions, is induced by biotic and abiotic factors in plants [58]. Therefore, the up-regulation of *LEA5* and *TLP1* in B-toxic *C. grandis* leaves might be an adaptive response.

Protein sodium-and lithium-tolerant 1 (SLT1) gene isolated from tobacco (*NtSLT1*) and *A. thaliana* (*AtSLT1*) has been implicated in mediating salt tolerance by regulating Na^+^ homeostasis *via* the calcineurin (CaN) and SPK1/HAL4 (SPK1/HAL4 which encodes a serine-threonine kinase) signal transduction [59]. Later, Antoine et al. [60] showed that rice *OsSLT1* had molecular chaperone activity *in vitro*, and that *OsSLT1* could be an important component of the cell immediate defenses against possible protein denaturation and aggregation. The down-regulation of *SLT1* (TDF #99_5) in B-toxic *C. grandis* leaves (Table 2) means that Na^+^ homeostasis or related processes mediated by *SLT1* are impaired in B-toxic *C. grandis* leaves.

Plant autophagy plays a role in various stress responses, pathogen defense, and senescence [61]. Xiong et al. [62,63] showed that AtATG18a was necessary for the formation of autophagosomes during nutrient stress and senescence in *A. thaliana* and that autophagy participated in the degradation of oxidized proteins under oxidative stress conditions in *Arabidopsis. AtATG18a* RNAi plants usually senesce earlier and have lower tolerance to various stresses including drought, salt and oxidative stresses compared with wild-type plants [61,63]. Our result showed that the transcript level of *transducin*/*WD40 domain*-*containing protein* (ATG18a, TDF #104_3) in *C. sinensis* leaves decreased in response to B-toxicity (Table 2), indicating that autophagy is impaired in *C. sinensis* leaves.

As shown in Table 2, B-toxicity down-regulated the expression of “cold-regulated” gene (cold regulated 314 thylakoid membrane 2, TDF # 109_1) in *C. sinensis* leaves and universal stress protein A-like protein (TDF #150_2) in *C. grandis* leaves (Table 2), indicating that B-toxicity might affect the tolerance of plants to other stresses.

### Leaf signal transduction

Here four genes involved in signal transduction were altered by B-toxicity (Table 2 and Figure 4). Evidence shows that that signal recognition particle 54 kDa protein (SRP54) plays important roles in chloroplast development [64,65]. The down-regulation of signal recognition particle 54 kDa protein 2 (TDF #182_2) in B-toxic *C. grandis* leaves (Table 2) means that the biosynthesis of Chl is impaired in these leaves. This agrees with our results that B-toxicity affected Chl more in *C. grandis* leaves than in *C. sinensis* ones (Figure 3E).

Increasing evidence shows that 14-3-3 proteins play an important role in plant stress responses [66,67]. The most direct evidence for the role of 14-3-3 proteins in stress responses comes from transgenic rice plants over-expressing *ZmGF14*-*6* encoding a maize 14-3-3 protein [68] and cotton plants over-expressing *Arabidopsis* 14-3-3λ [69]. These transgenic plants displayed enhanced tolerance to drought stress. Heterotrimeric GTP-binding proteins (G proteins, consisting of subunits G_α_, G_β_, and G_γ_) are signaling molecules required for various eukaryotic organisms. Joo et al. [70] observed that *A. thaliana* mutant plants losing the G_β_ protein were less tolerant to O_3_ damage than wild-type plants. Thus, the B-tolerance of *C. grandis* leaves might be down-regulated due to decreased transcript level of genes encoding 14-3-3 protein (TDF #108_2) and heterotrimeric GTP-binding protein subunit beta 1 (TDF #200_1) (Table 2).

In higher plants, the endogenous circadian clock is involved in the manipulation of different various cellular processes ranging from photosynthesis to stress responses [71,72]. It also confers plants with competitive advantages, including improved photosynthesis, growth and survival [71]. Nakamichi et al. [72] observed that A *PRR9*, *7* and *5* triple mutant of *Arabidopsis* had higher tolerance against drought, salt and cold stresses compared to wild type, demonstrating the involvement of the three genes in abiotic stress responses as negative regulators. The up-regulation of *pseudo*-*response regulator* 5 (*PRR5*; TDF #70_2) in B-toxic *C. sinensis* leaves (Table 2) agrees with the previous reports that *PRR5* was induced by cold treatment in apical shoots of cassava [73] and in *Arabidopsis* leaves [74]. Fukushima et al. [75] showed that PRR9, 7 and 5 negatively regulated the biosynthetic pathways of Chl, Car, ABA and α-tocopherol. This agrees with our results that B-toxici *C. sinensis* leaves had decreased concentrations of Chl a + b and Car (Figure 3E and H).

### Leaf cell transport

As shown in Table 2 and Figure 4, the number of differentially expressed TDFs involved in cell transport was far less in B-toxic *C. sinensis* leaves than in B-toxic *C. grandis* ones, meaning that cell transport is less affected in the former than in the latter, which agrees with our inference that *C. sinensis* leaves may tolerate higher level of B.

Most of the differentially expressed TDFs (TDFs #26_1, 124_3, 66_1, 97_1, 53_1, 210_1, 178_1, 49_3, 137_1, 63_1, 51_1, 250_2, 79_2 and 67_3) associated with cell transport were up-regulated in B-toxic *C. grandis* leaves except for AT5g24810/F6A4_20 (TDF #89_2), protein transport protein SEC61 γ subunit (TDF #6_1) and putative β-subunit of adaptor protein complex 3, PAT2 (TDF #249_2) (Table 2), indicating that cell transport might be enhanced in B-toxic *C. grandis* leaves. Plasma-membrane H^+^-ATPase plays a crucial role in the plant response to environmental stresses, such as salt stress, aluminum (Al) stress, P and potassium (K) deficiencies [76]. Wu et al. [77] reported that pumping of Ca^2+^ and Mn^2+^ by an endoplasmic reticulum-type Ca^2+^-ATPase (ECA1) into the endoplasmic reticulum was necessary for maintaining plant growth under calcium (Ca)-deficiency or Mn-toxicity. The P_IB_-ATPases (also known as heavy metal ATPases), which are involved in heavy metal transport across cellular membranes, play a crucial role in metal homeostasis and detoxification in plants [78]. Proton pump interactor 1 (PPI1), an interactor of plasma-membrane H^+^-ATPase, stimulates its activity *in vitro* [79]. The up-regulation of *PPI1* (TDF #97_1) in B-toxic leaves agrees with our data that the transcript level of *H*^+^-*ATPase 6* (TDF #26_1) in *C. grandis* leaves increased in response to B-toxicity (Table 2) and with the report that the expression of *PPI1* in potato tuber was up-regulated by salt stress and cold [79].

ATP-binding cassette (ABC) transporters are involved in metal ion efflux from the plasma-membrane. AtPDR8, an ABC transporter localized in the plasma-membrane of *A. thaliana* root hairs and epidermal cells, confers metal tolerance [80]. Our finding that the expression of ABC transporter G family member 40 (TDF #53_1) gene was up-regulated in B-toxic *C. grandis* leaves agrees with the reports that *AtPDR8* in *Arabidopsis* roots and shoots was induced when exposed to copper (Cu), cadmium (Cd) and lead (Pb) [80], and that ABC transporter G family member 40 gene and ABC transporter A family member 7 gene were induced in drought-sensitive and -tolerant genotypes of *Gossypium herbaceum*, respectively under drought stress [81]. However, the expression of AT5g24810/F6A4_20 (TDF #89_2) was down-regulated in B-toxic *C. grandis* leaves (Table 2).

Cu transporters (COPTs/Ctrs) are involved in the maintenance of Cu homeostasis in plants. Generally speaking, *COPTs*/*Ctrs* are up-regulated by Cu deprivation and down-regulated by Cu excess [82]. *COPT1* antisense *Arabidopsis* plants have decreased Cu level due to decreased Cu uptake and display sensitivity to Cu chelators [83]. The up-regulation of COPT (TDF #210_1) in B-toxic *C. grandis* leaves might play a role in the maintenance of leaf Cu homeostasis.

Plant cyclic nucleotide gated channels (CNGCs) paly a role in heavy metal homeostasis. Previous study showed that transgenic tobacco plants overexpressing a truncated *NtCBP4* (tobacco *CNGC*) had higher tolerance to Pb compared with wild type [84]. Chan et al. [85] reported that *cngc2 Arabidopsis* mutants were hypersensitive to increased soil Ca. However, transgenic tobacco plants overexpressing *NtCBP*4 were hypersensitivity to Pb [86]. B-toxicity-induced increase in transcript level of *CNGC1* (TDF #178_1) in *C. grandis* leaves (Table 2) agrees with the report that the expression of *AtCNGC2* was induced during *Arabidopsis* leaf senescence and *AtCNGC2* might be involved in programmed cell death [87].

Membrane traffic is not only required for plant normal cellular function and maintenance of cellular viability, but also plays an important roles in plant responses to the environment [88,89]. The transcript levels of genes [i.e., vacuolar-sorting receptor 3 (TDF #49_3), vacuolar protein-sorting-associated protein 37–1 (TDF #137_1), vesicle-associated membrane protein-associated protein (TDF #63_1), secY protein transport family protein (TDF #51-1), fat-free-like protein (TDF #250_2) and non-specific lipid-transfer protein (TDF #79_2)] involved in membrane traffic increased in B-toxic *C. grandis* leaves except for genes encoding protein transport protein SEC61 γ subunit (TDF #6_1) and putative β-subunit of adaptor protein complex 3, PAT2 (TDF # 249_2) (Table 2). This indicates that membrane traffic might be enhanced in B-toxic *C. grandis* leaves.

Plant sieve element occlusion (SEO) genes have been shown to encode the common phloem proteins (P-proteins) that plug sieve plates after wounding. Tobacco *SEO*-RNA interference lines were essentially devoid of P-protein structures and lost photoassimilates more rapidly after injury than control plants [90]. Therefore, the up-regulation of sieve element occlusion protein 1 gene (TDF #67_3) in B-toxic *C. grandis* leaves (Table 2) might be of advantage to prevent the loss of photoassimilates. Recently, Huang et al. observed that many electron-dense particles deposited near sieve plates of B-toxic *C. grandis* and *C. sinensis* leaves [13]. In conclusion, the up-regulation of cell transport in B-toxic *C. grandis* leaves might be an adaptive response of plants to B-toxicity.

By contrast, we isolated three down-regulated [i.e., putative β-subunit of adaptor protein complex 3, PAT2 (TDF #249_2), sugar transporter ERD6-like 5 (TDF #61_1) and metal tolerance protein (MTP, TDF #179_2)] and three up-regulated [i.e., sieve element occlusion protein 1 (TDF #67_3), kinesin-related protein (TDF #51_4) and bidirectional sugar transporter SWEET7 (TDF #36_2) TDFs from B-toxic *C. sinensis* leaves (Table 2). Generally speaking, cell transport might be not enhanced in B-toxicity leaves.

In plants, kinesins are involved in a variety of cellular processes including intracellular transport, spindle assembly, phragmoplast assembly, chromosome motility, MAP kinase regulation and microtubule stability [91]. Li et al. [92] reported that mutation of rice *BC12*/*GDD1* encoding a kinesin-like protein led to dwarfism with impaired cell elongation. Nishihama et al. [93] demonstrated that the expansion of the cell plate in tobacco plant cytokinesis required kinesin-like proteins (i.e., NACK1 and NACK2) to regulate the activity and localization of MAP kinase kinase kinase. Therefore, the up-regulation of *kinesin*-*like protein* (TDF #51_4) in *C. sinensis* leaves (Table 2) might be an adaptive responsive to B-toxicity. However, the transcript level of putative β-subunit of adaptor protein complex 3, PAT2 (TDF #249_2) in *C. sinensis* leaves decreased in response to B-toxicity (Table 2).

Plant SWEETs function as facilitators involved in the influx and the efflux of sugar into and out of cells [94]. We found that the expression level of *SWEET7* (TDF #36_2) in *C. sinensis* leaves increased in response to B-toxicity (Table 2), which agrees with the previous report that *SWEET15*/*SAG29* was enhanced in senescing *Arabidopsis* leaves [95]. However, the expression of gene encoding sugar transporter ERD6-like 5 (TDF #61_1), a passive facilitator for the diffusion of glucose across the tonoplast membrane, was down-regulated in B-toxic *C. sinensis* leaves (Table 2). This disagrees with the previous report that the expression of *AtESL1* (*ERD six*-*like 1*) was induced by various stresses including drought, high salinity and ABA in *Arabidopsis* plants [96].

MTPs are a subfamily of the cation diffusion facilitator (CDF) family found in plants. So far, most studied CDF family members confer heavy metal tolerance by affecting heavy metal efflux from the cytoplasm [97]. The down-regulation of *MTP* (TDF #179_2) in *C. sinensis* leaves (Table 2) means that the tolerance of plants to heavy metal might be reduced in B-toxic plants. This agrees with our previous report that the tolerance of *C. grandis* plants to Al-toxicity was higher under adequate B supply than under excess B [98].

### Leaf cell wall and cytoskeleton modification

Eleven TDFs associated with cell wall and cytoskeleton modification were altered by B-toxicity (Table 2 and Figure 4). O-methyltransferase (OMT) genes are involved in lignin biosynthesis. Fu et al. [99] showed that down-regulation of the caffeic acid 3-O-methyltransferase (COMT) gene in switchgrass lowered lignin level in whole tillers and stems of transgenic plants and enhanced forage quality. Transgenic *Leucaena leucocephala* plants expressing antisense *OMT* displayed decreased activity of OMT activity and concentration of lignin [100]. Therefore, the biosynthesis of lignin in B-toxic *C. grandis* and *C. sinensis* leaves might be reduced due to decreased expression of *COMT* (TDF #49_4) and *COMT3* (TDF #125_2) (Table 2). In addition, the biosynthesis of chitin in *C. grandis* and *C. sinensis* leaves and cellulose in *C. grandis* leaves might be down-regulated under B-toxicity due to the down-regulation of *chitinase* (TDF #10_3) and c*ellulose synthase* (TDF #249_4) (Table 2). These results demonstrated that B-toxicity might impair citrus cell wall metabolism, which agrees with the previous suggestion that leaf cupping, a specific visible B-toxic symptom in some species might be due to the inhibition of cell wall expansion, through disturbance of cell wall crosslinks [101]. However, the transcript levels of genes encoding OMT1 (TDF #33_3), LIM domain-containing protein (TDF #241_1), UDP-glucose flavonoid 7-O-glucosyltransferase (TDF #124_2), UDP-glucosyltransferase family 1 protein (TDF #3_3), limonoid UDP-glucosyltransferase (TDF #70_4) and putative glucosyltransferase (TDF #63_2) in *C. grandis* increased in response to B-toxicity (Table 2).

Evidence shows that lily LIM1 [87] and all *Arabidopsis* LIM domain proteins [102] participate in regulating actin cytoskeleton organization and dynamics. Tobacco LIM1 protein acts in the cytoplasm as an actin binding and bundling protein [103] and in the nucleus as a transcription factor regulating the expression of genes related to lignin biosynthesis [104]. Recently, Moes et al. [105] demonstrated the involvement of tobacco LIM2 in actin-bundling and histone gene transcription. The up-regulation of *LIM domain*-*containing protein* (TDF #241_1) in B-toxic *C. grandis* leaves (Table 2) agrees with the report that the expression of *LIM domain*-*containing protein* in *Physcomitrella patens* increased under cold acclimation [106].

Glycosyltransferases (GTs), which catalyze the formation of glycosidic bonds between donor sugars and acceptor molecules, participate in many aspects of a plant life, including cell wall biosynthesis [107,108]. In *Arabidopsis*, up to 10 or 12 GT2 family members form the cellulose synthase catalytic subunit and callose synthase gene families [108]. In plants, UDP-glucosyltransferases (UGTs) have been suggested to play important roles in keeping cell homeostasis, regulating plant growth and improving their tolerance to environmental stresses [109]. Overexpression of *UGT74E2* conferred tolerance to salinity and drought stresses in *A. thaliana* [110]. Transgenic tobacco plants overexpressiong *UGT85A5* exhibited enhanced salt tolerance [111]. Therefore, the up-regulation of UDP-glucose flavonoid 7-O-glucosyltransferase (TDF #124_2), UGT family 1 protein (TDF #3_3), limonoid UGT (TDF #70_4) and putative GT (TDF #63_2) genes in B-toxic *C. grandis* leaves (Table 2) might play a role in B-tolerance of plants. However, loss of function of a *UGT73B2* alone or in conjunction with *UGT73B1* and *UGT73B3* resulted in enhanced oxidative stress tolerance in *Arabidopsis*, whilst transgenic *Arabidopsis* plants overexpressing *UGT73B2* displayed decreased oxidative stress tolerance [112].

### Others

Overexpression of bacterial or plant gene encoding phytoene synthase (PSY), a key regulatory enzyme in Car biosynthesis, led to enhanced level of total Car in various higher plants [113,114]. Transgenic *Arabidopsis* plants overexpressing *PSY* from euhalophyte *Salicornia europaea* had higher tolerance to salt stress than wild type plants by enhanced photosynthetic efficiency and antioxidative capacity [115]. Cidade et al. [116] showed that ectopic expression of *PSY* from *Citrus paradisi* fruit conferred abiotic stress tolerance in transgenic tobacco, which was correlated with the increased endogenous ABA level and expression of stress-responsive genes. Our finding that B-toxic *C. grandis* leaves had lower transcript of *PSY* (TDF #229_4; Table 2) means that the biosynthesis of Car and the antioxidative capacity may be decreased in B-toxic leaves. This agrees with our data that B-toxicity affected Car more in *C. grandis* leaves than in *C. sinensis* one (Figure 3G) and the inference that *C. grandis* may tolerate lower level of B.

Strictosidine synthase (Str), a key enzyme in alkaloid biosynthesis, catalyzes the condensation of tryptamine and secologanin leading to the synthesis of numerous monoterpenoid indole alkaloids in higher plants [117]. The up-regulation of Str family protein gene (TDF #231_1) in B-toxic *C. sinensis* leaves (Table 2) agrees with the previous report that *Str* in *Catharanthus roseus* leaves was enhanced under dehydration, salt and UV stresses [117] and that B-toxicity decreased IAA level in *Triticum durum* seedlings [118], because the expression of *Str* was inhibited by auxin [119]. B-toxicity-induced up-regulation of Str family protein gene (TDF #231_1) also agrees with our reference that the AOS pathway and jasmonate level might be up-regulated in the B-toxic *C. sinensis* leaves due to decreased expression of fatty acid HPL gene (TDF #10_1) (Table 2), because *Str* has been shown to be induced by jasmonate [120]. By contrast, the expression of Str family protein gene (TDF #231_1) was down-regulated in B-toxic *C. grandis* leaves (Table 2), which agrees with the previous report that cold stress led to *Str* down-regulation in *C. roseus* leaves [117].

## Conclusions

B-toxicity affected *C. grandis* seedling growth, leaf CO_2_ assimilation, pigments, total soluble protein, MDA and P more than *C. sinensis*, indicating that *C. sinensis* have higher B-tolerance than *C. grandis* ones. Under B-toxicity, *C. sinensis* leaves accumulated more B than *C. grandis* ones, meaning that the former may tolerate higher level of B. Using cDNA-AFLP, we successfully isolated 67 up-regulated and 65 down-regulated TDFs from B-toxic *C. grandis* leaves, whilst only 31 up-regulated and 37 down-regulated TDFs from B-toxic *C. sinensis* ones. This indicates that gene expression is less affected in B-toxic *C. sinensis* leaves than in *C. grandis* ones, which might be associated with the fact that *C. sinensis* leaves can tolerate higher level of B. The higher B-tolerance of *C. sinensis* might be related to the findings that B-toxic *C. sinensis* leaves had higher expression levels of genes involved in photosynthesis, which might contribute to the higher photosynthesis and light utilization and less excess light energy compared to the B-toxic *C. grandis* ones, and in ROS scavenging, thus preventing them from photo-oxidative damage. In addition, B-toxicity-induced alteration in the expression levels of genes encoding inorganic PPase 1, AT4G01850 and methionine synthase differed between the two species, which might also contribute to the B-tolerance of *C. sinensis*. In this study, a total of 174 differentially expressed TDFs were isolated from two citrus species, only 26 TDFs presented in the two citrus, the remaining TDFs presented only in *C. grandis* or *C. sinensis*, demonstrating that the B-toxicity-responsive genes differ between the two citrus species. For example, cell transport were up-regulated in B-toxicity *C. grandis* leaves, whilst this did not occur in B-toxic *C. sinensis* ones.

## Methods

### Plant materials

This study was conducted from February to December, 2011 at Fujian Agriculture and Forestry University. Plant culture and B treatments were performed according to Han et al. [14]. Briefly, 5-week-old uniform seedlings of ‘Xuegan’ (*Citrus sinensis*) and ‘Sour pummelo’ (*Citrus grandis*) were transplanted to 6 L pots containing fine river sand. Plants, two per pot, were grown in a greenhouse under natural photoperiod at Fujian Agriculture and Forestry University. Eight weeks after transplanting, each pot was supplied every other day until dripping with nutrient solution containing 10 μM (control) or 400 μM (B-toxic) H_3_BO_3_ and 6 mM KNO_3_, 4 mM Ca (NO_3_)_2_, 2 mM NH_4_H_2_PO_4_, 1 mM MgSO_4_, 10 μM H_3_BO_3_, 2 μM MnCl_2_, 2 μM ZnSO_4_, 0.5 μM CuSO_4_, 0.065 μM (NH_4_)_6_Mo_7_O_24_ and 20 μM Fe-EDTA for 15 weeks. At the end of the experiment, fully expanded leaves from different replicates and treatments were used for all the measurements. Leaves were collected at noon under full sun and immediately frozen in liquid nitrogen and were stored at −80°C until extraction.

### Measurements of plant DW, root and leaf B, leaf P, total soluble protein, MDA and pigments

Ten plants per treatment from different pots were harvested and divided into their parts (roots and shoots). The plant parts were then dried at 75°C for 48 h and their DWs measured. B concentration in roots and leaves was assayed by ICP emission spectrometry after microwave digestion with HNO_3_ [121]. Leaf P concentration was measured according to Ames [122]. Leaf total soluble protein was measured according to Bradford [123] using bovine serum albumin as standard after being extracted with 50 mM Na_2_HPO_4_-KH_2_PO_4_ (pH 7.0) and 5% (w/v) insoluble polyvinylpyrrolidone. Extraction and determination of leaf MDA were performed according to Hodges et al. [124]. Chl, Chl a, Chl b and Car were assayed according to Lichtenthaler [125] after being extracted with 80 (v/v) actetone.

### Measurements of leaf gas exchange

Leaf gas exchange was measured using a CIARS-2 portable photosynthesis system (PP systems, Herts, UK) at ambient CO_2_ concentration under a controlled light intensity of 990–1010 μmol m^−2^ s^−1^ between 9:00 and 11:00 on a clear day. During measuring, leaf temperature and air relative humidity were 32.2 ± 0.2°C and 66.6 ± 0.8%, respectively.

### Leaf RNA extraction, cDNA synthesis and cDNA-AFLP analysis

Total RNA was extracted from ca. 300 mg of frozen mixed leaves from B-toxic and control plants of *C. grandis* and *C. sinensis* using Recalcirtant Plant Total RNA Extraction Kit (Centrifugal column type, Bioteke Corporation, China). There were three biological replicates for each treatment. Leave of 4–5 plants from different pots were mixed as a biological replicate. Equal amounts of leaves were collected from each plant. cDNA synthesis and cDNA-AFLP analysis were performed according to Zhou et al. [44].

### Validation of cDNA-AFLP data using qRT-PCR

Total RNA was extracted from the frozen leaves as described above. qRT-PCR analysis was performed according to Zhou et al. [44]. Specific primers were designed from the sequences of 16 differentially expressed TDFs using Primer Primier Version 5.0 (PREMIER Biosoft International, CA, USA). The sequences of the F and R primers used were listed in Additional file 3. Samples for qRT-PCR were run in 3 biological replicates with 3 technical replicates. Leave of 4–5 plants from different pots were mixed as a biological replicate. Relative gene expression was calculated using ddCt algorithm. For the normalization of gene expression, citrus *actin* (GU911361.1) was used as an internal standard and the leaves from control plants were used as reference sample, which was set to 1.

### Experimental design and statistical analysis

There were 20 pots (40 seedlings) per treatment in a completely randomized design. Experiments were performed with 3–10 replicates. Results represented the mean ± SE. Statistical analyses of data were carried out by ANOVA tests. Means were separated by the least significant difference test at *P* <0.05 level.

## Additional files

Additional file 1:
**Boron (B)-toxic symptoms on** ***Citrus grandis*** **and** ***Citrus sinensis*** **leaves.** 1: Control leaves of *C. grandis*; 2: B-toxic leaves of *C. grandis*; 3: Control leaves of *C. sinensis*; 4: B-toxic leaves of *C. sinensis*. Additional file 2:
**cDNA-AFLP profiles using one** ***Eco*** ***R I selective primer and eight*** ***Mes*** **I selective primers.** One *Eco*R I selective primer: *Eco*R I-GC; Eight *Mes* I selective primers: *Mes* I-GT, GA, TC, TG, TT, TA, AC and AG). 1: Control leaves of *Citrus grandis*; 2: B-toxicity leaves of *C. grandis*; 3: Control leaves of *Citrus sinensis*; 4: B-toxicity leaves of *C. sinenis*. Arrows indicate differentially expressed TDFs. Additional file 3:
**Specific primer pairs used for qRT-PCR expression analysis.**

## Abbreviations

ABC: ATP-binding cassette
ACP: Acyl carrier protein
AdoMet: S-adenosylmethionine
AO: Ascorbate oxidase
AOS: Allene oxide synthase
B: Boron
Car: carotenoid
CBS: Cystathionine-β-synthase
CDCP: CBS domain-containing protein
CDF: Cation diffusion facilitator
cDNA-AFLP: cDNA-amplified fragment length polymorphism
Chl: Chlorophyll
CNGC: Cyclic nucleotide gated channel
COMT: Caffeic acid 3-O-methyltransferase
COPT: Cu transporters
DW: Dry weight
GT: Glycosyltransferase
HPL: Hydroperoxide lyase
IF: Initiation factor
LEA5: Group 5 late embryogenesis abundant protein
MAP: Mitogen-activated protein
MATE: Multi-drug and toxic compound extrusion
MDA: Malondialdehyde
MTP: Metal tolerance protein
NUDX: Nudix hydrolases
OMT: O-methyltransferase
POR: Protochlorophyllide oxidoreductase
PPase: Pyrophosphatase
PPI1: Proton pump interactor 1
PRR: Pseudo-response regulator 5
PsbA: PSII 32 kDa protein
PsbP: PSII 23 kDa protein
PSI: Photosystem I
PSII: Photosystem II
PSY: Phytoene synthase
RNAi: RNA interference
ROS: Reactive oxygen species
Rubisco: RuBP carboxylase/oxygenase
RuBP: ribulose-1,5-bisphosphate
SBPase: Sedoheptulose-1,7-bisphosphatase
SEO: Sieve element occlusion
SLT1: Protein sodium-and lithium-tolerant 1
Str: Strictosidine synthase
TDF: Transcript-derived fragments
TLP: Thaumatin-like protein
UGT: UDP-glucosyltransferase.
