# Supplementary material for: cDNA-AFLP analysis reveals the adaptive responses of citrus to long-term boron-toxicity
Source: BMC Plant Biol. 2014 Oct 28;14:284. doi: 10.1186/s12870-014-0284-5 (PMC4219002; doi:10.1186/s12870-014-0284-5)
Supplement: Additional file 1: — Boron (B)-toxic symptoms on Citrus grandis and Citrus sinensis leaves. 1: Control leaves of C. grandis; 2: B-toxic leaves of C. grandis; 3: Control leaves of C. sinensis; 4: B-toxic leaves of C. sinensis. [file 12870_2014_284_MOESM1_ESM.doc]

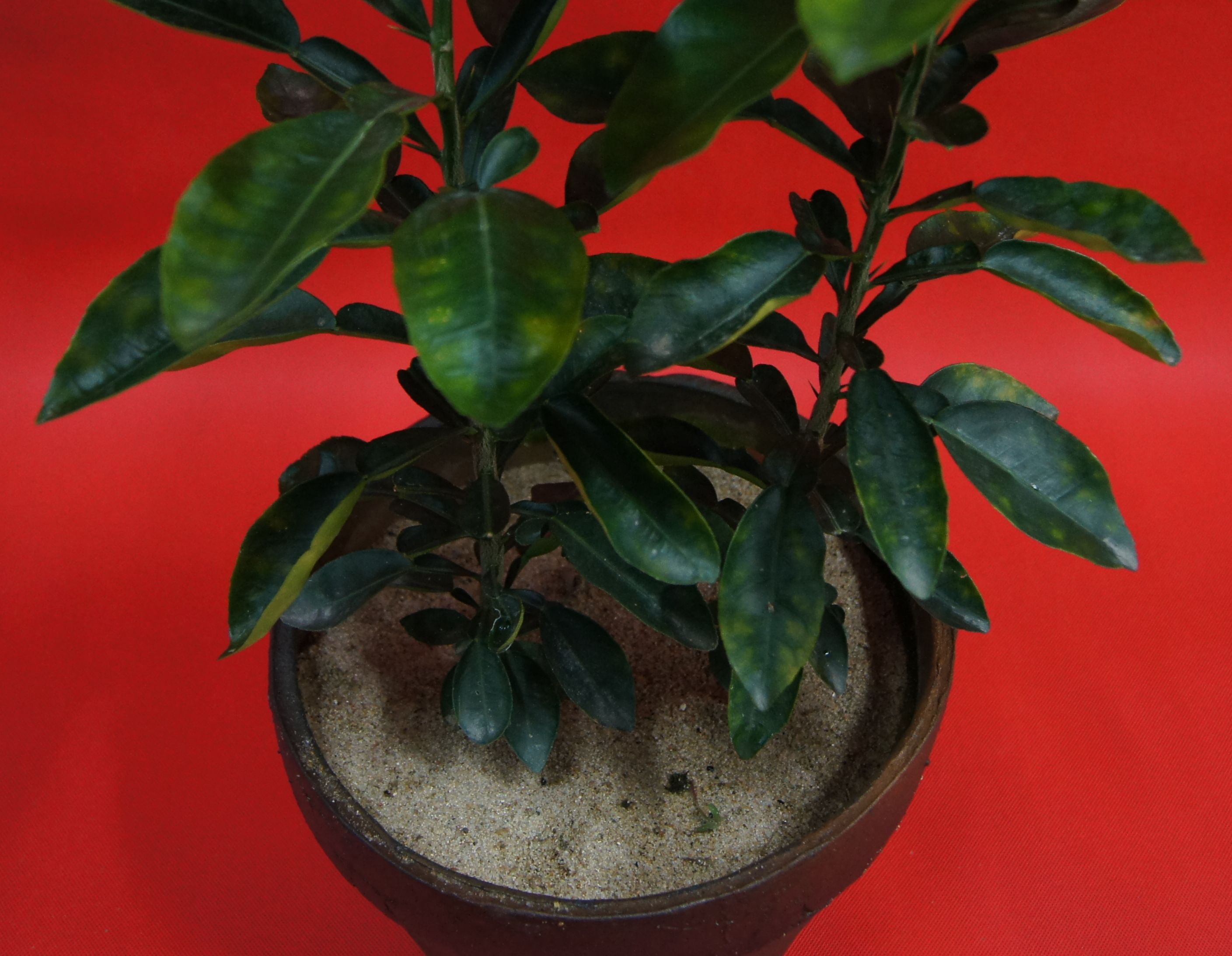


**A: *C. grandis***


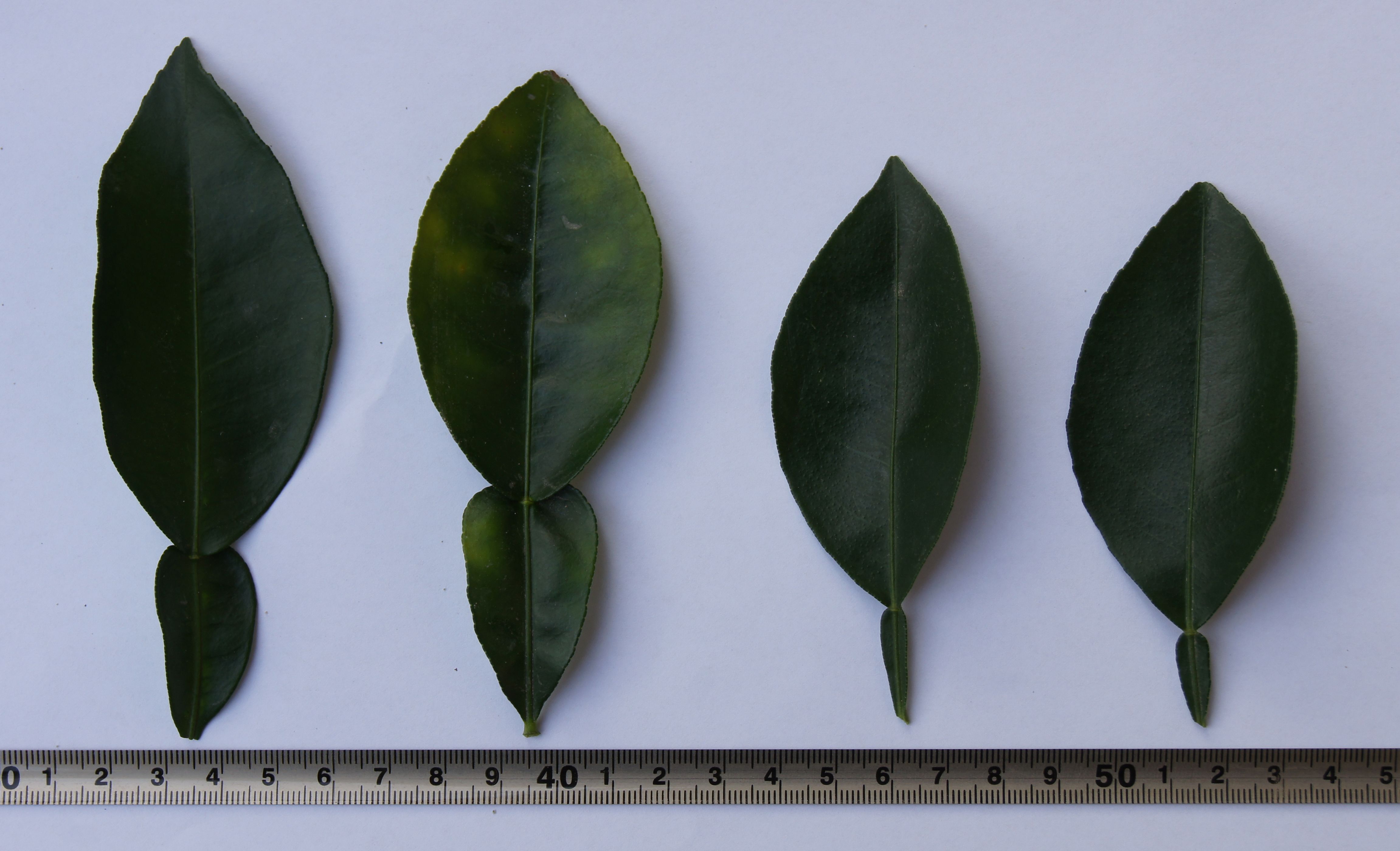


**1 2 3 4**

**B**

**Additional file 1: Boron (B)-toxic symptoms on *Citrus grandis* and *Citrus sinensis* leaves.** 1: Control leaves of *C. grandis*; 2: B-toxic leaves of *C. grandis*; 3: Control leaves of *C. sinensis*; 4: B-toxic leaves of *C. sinenis*.
