# Supplementary material for: cDNA-AFLP analysis reveals the adaptive responses of citrus to long-term boron-toxicity
Source: BMC Plant Biol. 2014 Oct 28;14:284. doi: 10.1186/s12870-014-0284-5 (PMC4219002; doi:10.1186/s12870-014-0284-5)
Supplement: Additional file 2: — cDNA-AFLP profiles using one Eco R I selective primer and eight Mes I selective primers. One EcoR I selective primer: EcoR I-GC; Eight Mes I selective primers: Mes I-GT, GA, TC, TG, TT, TA, AC and AG). 1: Control leaves of Citrus grandis; 2: B-toxicity leaves of C. grandis; 3: Control leaves of Citrus sinensis; 4: B-toxicity leaves of C. sinenis. Arrows indicate differentially expressed TDFs. [file 12870_2014_284_MOESM2_ESM.doc]

**aGC/GT GC/GA GC/TC GC/TG GC/TT GC/TA GC/AC GG/AG**


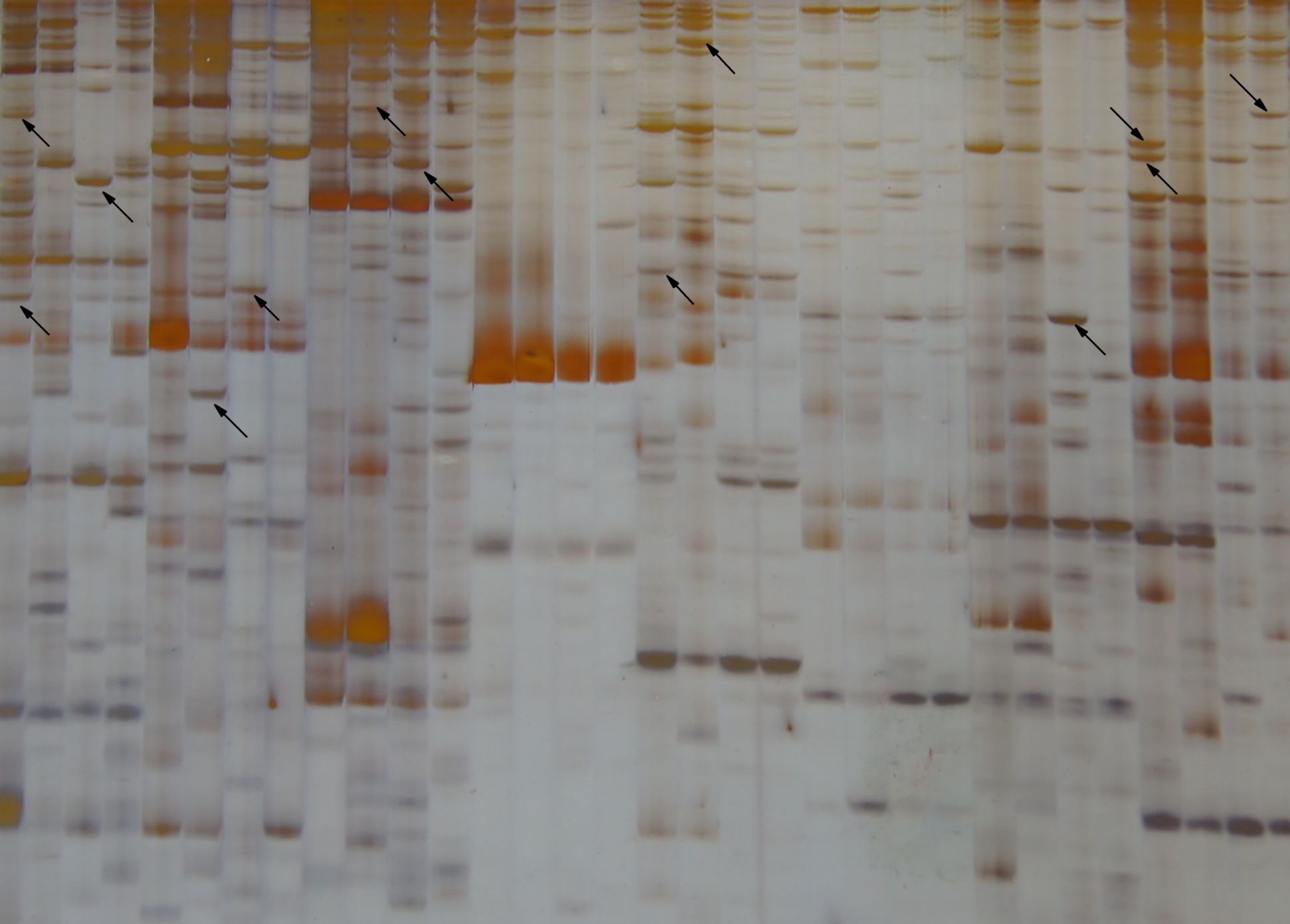


**1 2 3 4 1 2 3 4 1 2 3 4 1 2 3 4 1 2 3 4 1 2 3 4 1 2 3 4 1 2 3** **4**

**Additional file 2: cDNA**-**AFLP profiles using one** ***Eco*R I selective primer and eight** ***Mes*** **I selective primers.** One *Eco*R I selective primer: *Eco*R I-GC; Eight *Mes* I selective primers: *Mes* I-GT, GA, TC, TG, TT, TA, AC and AG). 1: Control leaves of *Citrus grandis*; 2: B-toxicity leaves of *C. grandis*; 3: Control leaves of *Citrus sinensis*; 4: B-toxicity leaves of *C. sinenis*. Arrows indicate differentially expressed TDFs.
