# Supplementary material for: cDNA-AFLP analysis reveals the adaptive responses of citrus to long-term boron-toxicity
Source: BMC Plant Biol. 2014 Oct 28;14:284. doi: 10.1186/s12870-014-0284-5 (PMC4219002; doi:10.1186/s12870-014-0284-5)
Supplement: Additional file 3: — Specific primer pairs used for qRT-PCR expression analysis. [file 12870_2014_284_MOESM3_ESM.doc]

**Additional file 3: Specific primer pairs used for qRT-PCR expression analysis.**

| TDF # or Gene | Forward primers (5´→3´) | Reverse primers (5´→3´) |
| --- | --- | --- |
| 10_1 | GGGGAGCGAGTTGTTGAGTT | GGCCGTGATCGAAGATGAGT |
| 10_3 | GCGATCCAATTCACCAAAGC | GAATCCCAAACTTGCCACAG |
| 23_2 | GCAGCAAATCAGCAACTAAGCC | TTCAGCCACATCCAGCCTCT |
| 26_1 | CTGCGATCCAATTCAGCACC | CATCCATACCAGCCATTTCCTC |
| 51_1 | CGGCCACTGTTTATGGTTGT | TTGCTATCGGTGAGGCTGTT |
| 73_1 | AAGGAGGTTGACAGAATGCC | AGTAAGCTGTTGCCGTGAGG |
| 87_1 | TGGATACCACAGGGCGAAGA | CAATTCCGCAACAGGCTTCA |
| 138_4 | CTCGTCTCACGGAGGTTAGGAA | CCTGAGTAAGGTCAGCAGCAA |
| 139_4 | CCCGATAAACTGTTACGGCTCA | TATCCCGCCGACTATTTGC |
| 143_2 | GAAAGGATGGGTGTACCGTGAG | GGCTTGGCAGCAATGAAACT |
| 145_1 | GCTAGGGACTGGCATGGAAA | CCAGGGAGGCATTGATTTGT |
| 148_2 | TATGGTGGCTACTGATTGGG | CTGGATCAACACGAGGGTAA |
| 171_2 | GAGCCAACGATGATGAGACG | GCTAAGGAGCCTACATTTACGG |
| 187_1 | AGTGGCCTGATCGACTTTGG | ATGAAGCAGTGGCGTTGGA |
| 195_1 | ACTGAGAAACTGTGGCAGGCATAAC | GCACTGAATTTGGTGGGAAGGA |
| 241_1 | TCTACAAGGAGCAACATCACCC | CAGCTTCCGACCATCATCAA |
| *Actin* | AGAACTATGAACTGCCTGATGGC | GCTTGGAGCAAGTGCTGTGATT |
